# Chordless cycle filtrations for dimensionality detection in complex networks via topological data analysis

**DOI:** 10.1038/s41467-026-72687-z

**Published:** 2026-05-06

**Authors:** Aina Ferrà Marcús, Robert Jankowski, Meritxell Vila-Miñana, Carles Casacuberta, M. Ángeles Serrano

**Affiliations:** 1 Departament de Matèmatiques i Informàtica, Universitat de Barcelona, Barcelona, Spain; 2 Departament de Física de la Matèria Condensada, Universitat de Barcelona, Barcelona, Spain; 3 Universitat de Barcelona Institute of Complex Systems (UBICS), Universitat de Barcelona, Barcelona, Spain; 4 Faculty of Electrical Engineering, Mathematics and Computer Science, Delft University of Technology, Delft, Netherlands; 5 Center for Complex Networks and Systems Research, Luddy School of Informatics, Computing, and Engineering, Indiana University, Bloomington, IN USA; 6 ICREA, Barcelona, Spain

**Keywords:** Applied mathematics, Complex networks

## Abstract

Many complex networks, ranging from social to biological systems, exhibit structural patterns consistent with an underlying hyperbolic geometry. Revealing the dimensionality of this latent space can disentangle the structural complexity of communities, impact efficient network navigation, and fundamentally shape connectivity and system behavior. We introduce a topological data analysis weighting scheme for graphs based on chordless cycles to estimate network dimensionality in a data-driven way. We further show that the resulting descriptors can effectively estimate network dimensionality using a neural network architecture trained on a synthetic graph database constructed for this purpose, which requires no retraining to transfer effectively to real-world networks. Thus, by combining cycle-aware filtrations, algebraic topology, and machine learning, our approach provides a robust and effective method for uncovering the hidden geometry of complex networks and guiding accurate modeling and low-dimensional embedding.

## Abstract (web summary)

Here, authors infer the latent hyperbolic dimensionality of complex networks using a cycle-aware topological weighting scheme and a neural network model trained on synthetic graphs, enabling robust, retraining-free estimation and improved understanding of hidden network geometry.

## Introduction

The rapid growth of data has created significant challenges in science and technology. Large datasets from fields such as biology (e.g., gene expression and protein interactions), social networks (e.g., agent behavior), and physics (e.g., cosmological simulations) often contain rich structural information hidden in their complex nature. Capturing this information requires tools capable of detecting patterns in data. Topological Data Analysis (TDA)^1–4^ is one such approach that uses ideas from topology to identify features that persist across scales.

In TDA, scales are usually defined through filtrations, a systematic way to build a sequence of simplicial complexes that encode the geometric and topological structure of a dataset across multiple levels of resolution. Central to the analysis of such simplicial complexes is persistent homology^2,3^, a main TDA computational method that tracks the emergence, persistence, and disappearance of topological features —such as connected components or loops— in a filtered simplicial complex as the filtration parameter evolves. In this context, graphs can be treated as simplicial complexes and filtered by assigning weights to their nodes and/or edges.

The success of persistent homology depends critically on the choice of a filtration and the topological features of interest, and these choices depend on the problem being addressed. Not every filtration or feature descriptor is suitable for every problem, underscoring the importance of designing suitable TDA pipelines to achieve meaningful results. The triad filtration-feature-framework (FFF) embodies an intertwined relationship that forms the foundation for effectively leveraging persistent homology. However, filtration schemes based on a variety of quantifiers, such as Forman–Ricci curvature^5^ or betweenness centrality^6^, are sometimes applied indiscriminately to data without considering the specifics of the selected topological features and the nature or framework of the problem at hand. This work emphasizes the importance of aligning the FFF triad by addressing dimensionality detection in complex networks, thereby bridging the gap between complex network theory and TDA. Simplicial complexes associated with complex networks have emerged as powerful representations in network science^7–11^, offering insights into higher-order structures beyond pairwise interactions. Moreover, persistent homology of filtered simplicial complexes has been applied to the study of complex networks in general^12–17^ and to neuroscience in particular^18–21^.

The problem addressed, the detection of the effective dimensionality of the space in which a complex phenomenon unfolds, is a recurring theme across the sciences. In statistical physics, the spatial dimension strongly constrains scaling laws and often determines the universality class of critical, extended systems in which multiple length scales are relevant. Even in the Ising model —the simplest widely used model of collective behavior and phase transitions, in which binary spins tend to align with their neighbors— dimensionality has a strong impact on critical behavior^22,23^. Diffusion processes are also often studied through random walks on *D*-dimensional lattices, where the dimension controls return probabilities, exploration rates, and typical first-passage times^24–26^. In other areas of physics, such as string-theory settings, extra dimensions beyond the observed three are needed, but remain inaccessible because they are tightly compactified or because observable dynamics are confined to a lower-dimensional subset^27^. A similar idea appears in computer science: although data and interactions may live in a formally high-dimensional space, relevant configurations typically occupy a much smaller effective subspace, with important implications for both structure and dynamics^28,29^. Dimensionality is also crucial in biology: transport and diffusion of molecules, encounter rates, and search processes depend sensitively on effective dimension^30,31^, and the structure of regulatory, neuronal, and ecological networks can enforce low-dimensional or genuinely multiscale dynamics, with different dimensions governing local versus global behavior^32^. Thus, the effective dimensionality of complex phenomena has direct implications for structural properties, dynamical regimes, and the mechanisms that dominate robustness, controllability, and criticality in complex systems.

In network science, several non-equivalent definitions of dimensionality that probe different aspects of structure and dynamics have been proposed in the literature. Topological shortest pahts have been used to define chemical or fractal (box-counting) dimension^33–37^, including networks with explicit geometrical embedding^38^; related to this, metric dimension is defined as the smallest number of nodes required to identify all other nodes uniquely based on shortest path distances^39^; correlation dimension is computed from network trajectories of random walkers^40^; and spectral dimension depends on the spectral properties of the graph Laplacian^41,42^. The problem has also been addressed within the framework of network geometry^43^. A model-driven approach^44^ leverages the geometric ${{\mathbb{S}}}^{D}/{{\mathbb{H}}}^{D+1}$ model^45,46^, which reproduces the observed connectivity of real networks, to reveal their intrinsic dimensionality in a latent hyperbolic space, where nodes are more likely to be connected if they are closer to each other. The real network’s specific dimension is determined by projecting the frequencies of chordless cycles of varying lengths onto the background model’s statistical configuration space. This technique revealed ultra-low-dimensional structures in real networks previously masked by apparent high-dimensionality^44^. This includes, for instance, tissue-specific biomolecular networks being extremely low-dimensional, brain connectomes being close to the three dimensions of their anatomical embedding, and social networks and the Internet, requiring slightly higher dimensionality. Within the same modeling framework, an alternative method for determining network dimensionality uses an embedding technique, named *D*-Mercator^47^, to produce multidimensional maps of real networks in (*D* + 1)-hyperbolic space whose dimensionality strongly shapes network properties, including community diversity^46,48^. The maps are used to estimate intrinsic dimensionality based on navigability and community structure, yielding results consistent with the statistical configuration space approach.

Despite this broad relevance, the concept of network dimensionality remains largely unexplored within TDA. In this work, we propose a method for dimensionality detection in complex networks based on TDA, which complements a trilogy in combination with the configuration-space and embedding methods used in ref. ^44^ and ref. ^47^, respectively. The dimensionality detection task, crucial for understanding the intrinsic geometry of data, showcases how tailored filtrations and feature selection can enhance the ability of persistent homology to analyze and interpret complex data for specific purposes. Specifically, we introduce a chordless cycle filtration scheme and use it to compute extended persistence of cycles, as the topological descriptor that best captures the distribution of cycles in synthetic and real networks to predict their dimensionality.

Moreover, we propose a data-driven approach to estimate network dimensionality by training a neural network on nearly 800 000 synthetic networks. A multilayer perceptron accurately estimates dimensionality and transfers effectively to real-world networks, generalizing and adapting to new data without retraining. Ablation experiments demonstrate that TDA features play an important role in this task, even when combined with average cycle densities and degree-related graph features.

## Results

Our approach to estimating network dimensionality using persistent homology is twofold. In ref. ^44^, it has been shown that measuring the intrinsic dimensionality of a complex network is possible by computing profiles of structural properties that are sensitive to dimensionality. These properties are the densities of chordless cycles of sizes three (triangles), four (squares), and five (pentagons), but the method required generating ensembles of synthetic networks for each candidate. Here, we instead focus on persistence summaries based on cycle density filtration and show that these descriptors reliably detect latent dimensionality. We then leverage the descriptors to develop a supervised machine-learning model, trained on a large database of synthetic graphs with known dimensions and controlled properties, to predict the intrinsic dimension of an input network.

### Persistence descriptors for dimensionality estimation

Many real networks share universal properties, such as sparsity, heavy-tailed degree distributions, the small-world effect, high clustering coefficients, and self-similarity. These properties can be captured by a simple geometric framework^49^ using the ${{\mathbb{S}}}^{1}/{{\mathbb{H}}}^{2}$ model^45,50^, which combines a popularity coordinate —controlling node degrees— with a similarity coordinate that represents all other attributes influencing network connectivity. This model can be generalized to a *D*-dimensional similarity space, yielding the ${{\mathbb{S}}}^{D}/{{\mathbb{H}}}^{D+1}$ model^45,46^; see Section 4 for more details. These models have been used to determine the dimensionality of real networks by analyzing their cycle profiles^44^. Here, however, we propose to measure their persistence descriptors instead.

The goal of persistent homology is to compute topological features of a space equipped with a filtration. In the context of graphs, one can define filtrations by assigning suitable weights to nodes or edges. A common choice is the degree filtration on nodes; however, it has been shown that the degree filtration is less expressive for some graph learning tasks than other, motif-based, filtrations^1^. In this work, we propose an edge weighting scheme based on densities of chordless cycles. A chordless cycle is defined as a closed edge path in which no two non-consecutive nodes are connected by an edge.

Our goal is to determine an optimal value of *D* for the ${{\mathbb{S}}}^{D}/{{\mathbb{H}}}^{D+1}$ model corresponding to a real network, in which *γ* and *β* —the parameters of the model controlling the scale-free degree distribution and the clustering coefficient, respectively— are usually unknown. In our data-driven method, optimality is defined in the same way as in ref. ^44^, by picking the dimension of a closest point in the configuration space of synthetic surrogates generated from the given real network, using *D*-dimensional geometric randomization (D-GR), as described in Section 4.1, with different values of *γ*, *β*, and *D*. The D-GR model, originally proposed for *D* = 1^51^, works on the observed sequence of node degrees and rewires the network to maximize the likelihood that the new topology is generated by the ${{\mathbb{S}}}^{D}/{{\mathbb{H}}}^{D+1}$ model.

Thus, for each complex network *G*_0_, whose dimensionality is to be estimated, we generated an ensemble of synthetic surrogates *G*_1_, …, *G*_*n*_ using the ${{\mathbb{S}}}^{D}$ model with a range of values of *D* and a range of values of the clustering coefficient *β* until *β* = 6*D*. Densities of edge triangles, chordless squares, and chordless pentagons were then computed as specified in Section 4.2 for each surrogate in the ensemble and for the target network. Therefore, each network *G* yields three weighted graphs (*G*, *w*_*t*_), (*G*, *w*_*s*_) and (*G*, *w*_*p*_), where the edge weights *w*_*t*_, *w*_*s*_, *w*_*p*_ are the densities of triangles, squares, and pentagons, respectively. The mean value of *w*_*t*_ over all the edges of a graph is denoted by *C*_*t*_, and similarly for squares and pentagons.

**Fig. 1:**
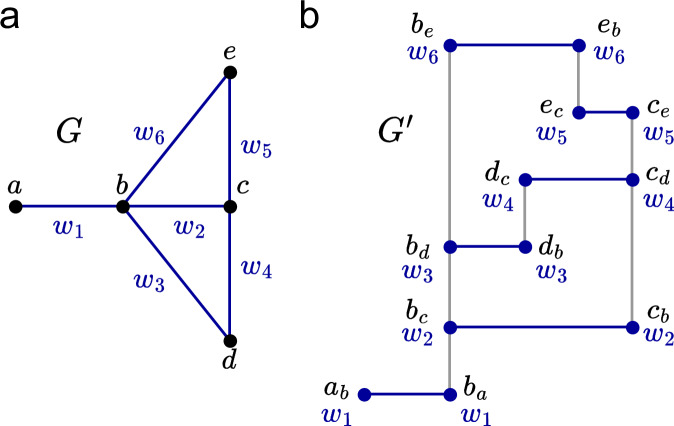
Extended persistence for an edge-weighted graph filtration. Topologically equivalent graphs equipped with (**a**) an edge weighting and (**b**) a node weighting, with the same extended persistence barcode. Assuming that *w*_1_ < ⋯ < *w*_6_, there are two generating cycles with birth-death coordinates (*w*_4_, *w*_2_) and (*w*_6_, *w*_3_), yielding a total extended persistence of ∣*w*_2_ − *w*_4_∣ + ∣*w*_3_ − *w*_6_∣. See Section 4.2 for details.

Topological features of graphs equipped with a filtration defined by edge weights were computed using persistent homology, a tool from algebraic topology that describes shape characteristics of many kinds of data (Section 4.2). In this work, persistence refers specifically to the evolution of cycles along the values of a given filtration. However, cycles in graphs have infinite persistence, since there are no higher-dimensional simplices to eventually fill them. We therefore computed extended persistence of cycles, defined as the difference between the largest and smallest weights among the simplices forming a closed path (see Fig. 1). Since we focus on graphs equipped with edge weightings only, we introduce in this article a technique for replacing a given edge-weighted graph with a larger, topologically equivalent graph, that carries weights on nodes and on the original edges in a way that is consistent with both sublevel and superlevel filtrations. This is illustrated in Fig. 1 and explained in more detail in Section 4.2.

**Fig. 2:**
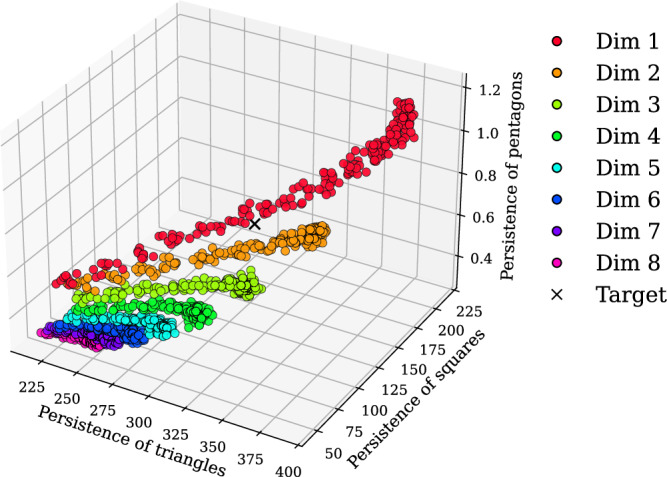
Total persistence values obtained from the human connectome. 3D view of a point cloud representing an ensemble of 1330 surrogates of the Human2 network (connectome of the human brain, including one hemisphere) in the configuration space of total persistence computed from three chordless cycle densities (triangles, squares, and pentagons). Points are colored by dimension. The target network is marked with a black cross.

Total extended persistence was used as a topological descriptor, resulting in a feature vector (*TP*_*t*_, *TP*_*s*_, *TP*_*p*_) for each network. A representation of the configuration space is shown in Fig. 2, where each point corresponds to a surrogate graph. Points are colored by the corresponding dimension. The target network from which surrogates were generated has *D* = 1 and is marked with a black cross.

Plausibility of an association between total extended persistence of cycles in a graph and its embedding dimensionality is illustrated in Figs. S1, S2, and S3, which show that dimensionality is inversely associated with total extended persistence values. A negative correlation is more evident in the case of squares and pentagons, with respective Pearson coefficient values of *ρ*_*s*_ = − 0.3140 and *ρ*_*p*_ = − 0.2433, versus *ρ*_*t*_ = − 0.0522 for triangles, calculated using a sample of size 50 000 from the SYNNET database (Section 2.2).

**Fig. 3:**
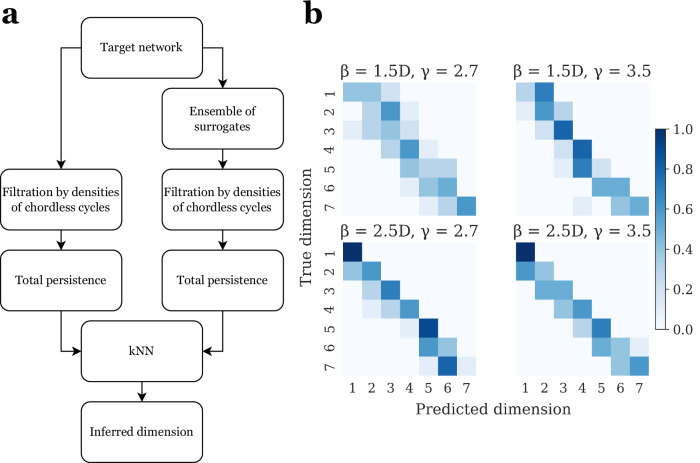
Dimensionality estimation with persistence descriptors. **a** Pipeline of our first method and **b** confusion matrices. Given a target network, we generate an ensemble of surrogates and, for each of them (including the given network), we compute densities of chordless cycles. We equip the graphs with these weightings and perform a topological analysis using extended persistence. For each network, we compute the total persistence of cycles and use a kNN classifier in the configuration space to infer a dimensionality D^*^ for the real network. The upper two confusion matrices show results obtained with synthetic networks for *β* = 1.5 *D* and two different values *γ* = 2.7 and *γ* = 3.5. This choice of *β* corresponds to the small-world phase even if *γ* > 3. The lower two confusion matrices show results for *β* = 2.5 *D*; in this case, networks with *γ* > 3 are large worlds. In each matrix, the rows correspond to the true dimension *D*, and the columns correspond to the inferred dimension D^*^. Thus, the *j*-th box in the *i*-th row shows the fraction of *i*-dimensional networks that were classified as *j*-dimensional. The darker the color, the greater the number. Each matrix is evaluated with 70 synthetic networks.

To estimate the target network's dimensionality, a *k*-nearest neighbors (kNN) classifier was used. The classifier identifies the *k* surrogates closest to the target network in the surrogate configuration space (*TP*_*t*_, *TP*_*s*_, *TP*_*p*_) by minimizing Euclidean distance. The value of *k* was not fixed, but it was determined within each ensemble of surrogates by finding the value of *k* with the highest accuracy in classifying each surrogate in the ensemble. Specifically, the inferred dimension D^*^ of the target network *G*_0_ maximizes the weighted frequency $f(D)={\sum }_{i=1}^{k}{\omega }_{i}\,{\delta }_{{D}_{i},D},$ where the normalized weights are inversely proportional to the distance between the real network and the *i*-th surrogate *G*_*i*_ in the (*TP*_*t*_, *TP*_*s*_, *TP*_*p*_) space, and ${\delta }_{{D}_{i},D}$ is the Kronecker delta function^44^. A schematic pipeline summary of the suggested methodology is shown in Fig. 3a.

The closeness of the kNN approximation was measured using a congruency index that reflects the fidelity of the surrogates with respect to the target network. The index is defined as the ratio $\rho={d}_{0}/\overline{d}$, where *d*_0_ is the distance between the point in configuration space corresponding to the target network and its closest neighbor, and $\overline{d}$ is the average of the distances between each surrogate point and its closest neighbor. Hence, the value of *ρ* is large when the point cloud is clustered, while the target network falls far away from the clusters. For comparability reasons, it is convenient to provide values of 1/*ρ*, as in Table 1, since, in most cases, 1/*ρ* takes values between 0 and 1, with values closer to one indicating higher fidelity between the target network and its surrogates.

**Table 1:** Inferred dimensionality of real-world networks

Network	Domain	Densities		TDA		DIMNN
		dim	1/*ρ*	dim	1/*ρ*	dim
Human2-C	Biological (Connectome)	3	0.07	1	0.21	2
Human-M	Biological (Metabolic)	3	0.19	4	0.46	3
Cargoships	Economic (Trade)	3	0.10	2	0.09	5
Bible-CO	Informational (Language)	4	0.05	1	0.04	5
Jazz-CA	Social (Collaboration)	2	0.06	2	0.41	2
EUEmail	Social (Communication)	2	0.09	1	0.28	2
Friends-ON	Social (Communication)	6	0.06	6	0.19	7
Friends-OFF	Social (Offline)	8	0.72	8	0.28	10

Comparison of the inferred dimension between three different methods: (1) Mean densities of chordless cycles; (2) TDA: Persistent homology from chordless cycle density filtrations; (3) DIMNN: A neural network trained on a database of synthetic networks (introduced in Section 2.2). The 1/*ρ* values are inverses of the congruency indices; higher values correspond to a closer match between the real network and its surrogate models.

As an evaluation step, the performance of the chordless cycle density filtration was tested on synthetic target networks generated using the ${{\mathbb{S}}}^{D}$ model for specific values of *D* (from 1 to 7), with different degree heterogeneities by varying the exponent (*γ* = 2.7 and *γ* = 3.5), and two values of inverse temperature (*β* = 2.5 *D*, corresponding to the high clustering regime, and *β* = 1.5 *D*, corresponding to the low clustering regime). For each combination (*D*, *γ*, *β*), ten synthetic target networks were generated, and the above method was applied to each of them to infer a dimensionality D^*^. Inferred dimensionalities were compared with the original dimension *D* from which the target synthetic network was generated. Confusion matrices for visualizing the inference method's performance are shown in Fig. 3b. Our predictions show some small confusion around contiguous values of *D* along the diagonal, especially for low values of *β* and *γ*, but this is expected given the high heterogeneity of the degree distribution.

Estimated dimension values for selected real-world target networks using TDA are shown in Table 1. The resulting values were compared with those obtained by implementing the method described in ref. ^44^, using the configuration space of mean cycle densities (*C*_*t*_, *C*_*s*_, *C*_*p*_), which have been recalculated using the D-GR procedure. Inverse values of the congruency index *ρ* are shown. As additional information, 2D projections of the configuration space (*TP*_*t*_, *TP*_*s*_, *TP*_*p*_) for the selected real-world networks are displayed in Figs. S1 and S2.

### Dimensionality estimation using neural networks

The method described in the previous section and the approach from ref. ^44^ rely on a large set of surrogate networks. For each new real network, one needs to generate a set of synthetic networks, compute their properties, and use a classifier to detect the dimension. As a consequence, when a new network is considered, the entire pipeline must be repeated. In this section, we propose an alternative based on a neural network that, once trained, can estimate dimensions directly, even for very large networks, generalizing and adapting to new data without retraining.

Neural networks excel for such tasks, since our aim is to approximate an unknown function yielding dimensionality values from a collection of predictors, including mean chordless cycle densities and/or persistence of corresponding filtrations. For this purpose, we created a database of synthetic complex networks, called SYNNET and used it to train a neural network, named DIMNN, to estimate the dimensionality of real-world networks. The main advantage of this method is that training in our database is performed only once and independently of specific target networks. This avoids the need to generate surrogates for each case under study. In total, we produced 792,000 synthetic networks generated from the ${{\mathbb{S}}}^{D}$ model (see Section 4.3 for more details). An 80–20% training-validation split was used.

For each synthetic network in the database, chordless cycle densities were computed as in Section 4.2 and averaged over all edges, as well as total persistence values obtained from the corresponding filtrations, and, additionally, the first moment and the normalized second moment of the degree distribution (i.e., the expected square divided by the square of the expected value). The normalized second moment is sensitive to degree fluctuations, particularly at very high degrees: in highly heterogeneous networks, the ratio is large, whereas in homogeneous networks it is close to 1. Hence, the normalized second moment tends to correlate with *γ*. Likewise, the average density of triangles *C*_*t*_ approximates the inverse temperature *β*.

Other relevant descriptors of complex networks that we integrated in our feature vectors are minimum and maximum degree, and average neighbor degree, which is denoted by 〈*k*_nn_〉 (see Section 4.2). This is a measure used to quantify degree-degree correlations in a graph, that is, how the degree of a node relates to the degrees of its neighbors.

**Fig. 4:**
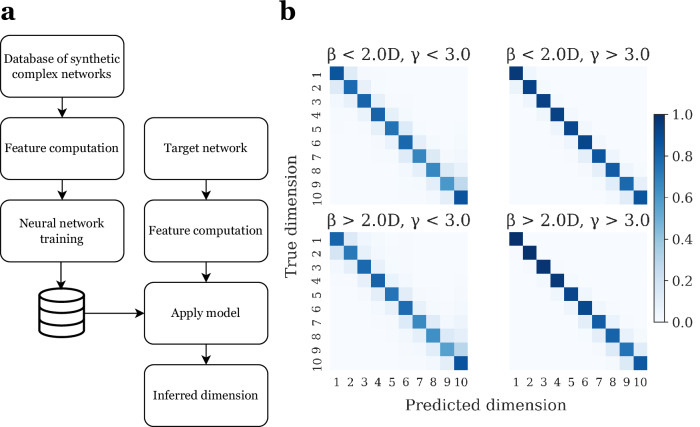
Dimensionality estimation using DIMNN. **a** Pipeline of method and **b** confusion matrices. A multilayer perceptron is trained on a database of synthetic complex networks, with features coming from filtrations by densities of chordless cycles concatenated with averages of chordless cycle densities and degree-related graph features. The parameters of the trained model are stored and the model is applied to target networks. Confusion matrices show results for the four combinations of *β* < 2*D* versus *β* > 2*D*, and *γ* < 3 versus *γ* > 3, from a range of values 1.2 *D* to 5.0 *D* for *β* and 2.2 to 5.0 for *γ*. In each matrix, the rows correspond to the true dimension *D* and the columns correspond to the inferred dimension D^*^.

Feature vectors consisting of the number of nodes, average degree, normalized second moment of the degree distribution, minimum and maximum degrees, average neighbor degree, mean chordless cycle densities *C*_*t*_, *C*_*s*_, *C*_*p*_, and total persistences *TP*_*t*_, *TP*_*s*_, *TP*_*p*_ of the corresponding filtrations were fed into a residual multilayer perceptron (see Section 4.3 for details). Our pipeline is shown in Fig. 4a. The highest classification accuracy obtained with DIMNN for estimating the dimensionality in the range *D* = 1 to *D* = 10 was 83.00% on the validation set with the full feature vector. Confusion matrices are shown in Fig. 4b.

**Fig. 5:**
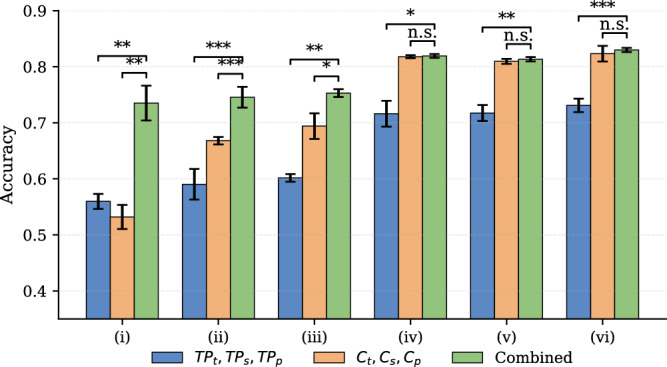
Validation accuracies of DIMNN using the SYNNET database. Blue color: Accuracies obtained using total persistences *TP*_*t*_, *TP*_*s*_, *TP*_*p*_, plus cumulative features; Orange color: Accuracies using average cycle densities *C*_*t*_, *C*_*s*_, *C*_*p*_, plus cumulative features; Green color: Accuracies obtained combining total persistences and mean cycle densities plus cumulative features. Successive columns correspond to incorporating one after the other the following additional features into the model: (i) no added features; (ii) number of nodes *N*; (iii) number of nodes and average degree 〈*k*〉; (iv) number of nodes, average degree, and normalized second moment 〈*k*^2^〉/〈*k*〉^2^; (v) number of nodes, average degree, normalized second moment, minimum degree ${k}_{\min }$ and maximum degree ${k}_{\max }$; (vi) number of nodes, average degree, normalized second moment, minimum degree, maximum degree, and mean average neighbor degree 〈*k*_nn_〉. Results are averaged over five realizations of DIMNN, and error bars indicate standard deviation. Horizontal brackets indicate paired comparisons between the combined model and each baseline; significance corresponds to paired *t*-tests with *p* < 0.05 (*), *p* < 0.01 (**), and *p* < 0.001 (***), while “n.s.” indicates a non-significant difference.

We also performed an ablation study, selecting subsets of the feature vector for both training and predictions. In Fig. 5, we show how the performance changes as a set of features is added at a time. The lowest accuracy is obtained when we use only the vector of mean chordless cycle densities with no other added features. Once degree-related properties are appended to the feature vector —e.g., minimum, maximum, and average degrees—, the accuracy increases, and incorporating topological information improves the accuracy further. In fact, combining total persistence with mean chordless cycle densities maximizes the performance. In Table S2, we show mean validation accuracies as well as the number of epochs and total training times, and a corresponding heatmap is provided in Fig. S6.

Next, we show the results of applying the trained DIMNN to detect the dimensionality of real-world complex networks. We analyzed the ten networks from the previous section and predicted their dimensions. Table 1 compares the inferred dimension across three different methods. One can observe that the predictions do not vary significantly by more than one or two dimensions in most cases, highlighting the robustness of the trained neural network. We incorporated another dataset of real complex networks^52^. First, we filtered out bipartite and temporal networks and restricted to networks with topological properties aligned with the training dataset (Fig. S8). Ranges of SYNNET database features are shown in Table S1. In total, we collected 53 new networks, whose properties are summarized in Tables S4 and S5.

**Fig. 6:**
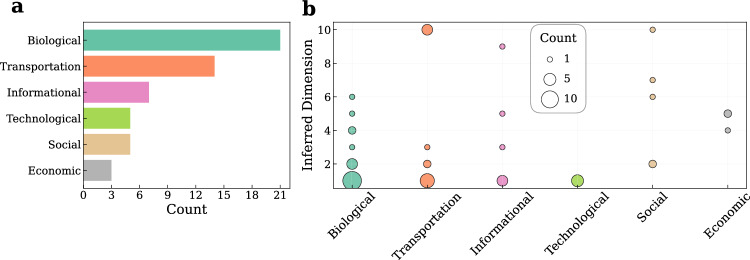
Dimensionality estimation of real networks with DIMNN. **a** Number of networks for each network domain. **b** Inferred dimension of real networks using DIMNN, grouped by network domain. The marker size indicates the number of networks.

In Fig. 6a, we group the dataset by domain, showing that the Biological and Transportation categories together account for more than half of all networks. We then apply our trained neural network to predict the intrinsic dimensions of these real-world networks (see Fig. 6b). Biological networks span a wide range of dimensions (from 1 through 6), whereas all networks in the Software domain lie at dimension 1. A closer look at subdomains (Fig. S9) reveals that web graphs —an Informational subdomain— also have dimension 1, while language networks (also Informational) appear at dimensions 5 and 9.

Subsequently, to enhance confidence in DIMNN's predictions, a complementary neural network model was trained. The base architecture was the same as for DIMNN, but the loss function was changed to mean squared error (MSE) to transform a classification task into a regression task. Thus, contrary to DIMNN, the output range of the regressor was not restricted to the interval [1, 10]. Since regression yields continuous values, each prediction was rounded to the nearest integer. We use the term pseudo-accuracy to denote the accuracy obtained by the regressor after rounding its predictions to integers. Moreover, residual connections were removed.

The regressor model exhibits behavior very consistent with that of DIMNN on synthetic networks (Fig. S7). The percentage of agreement between the two models on the validation set is 79.33% if total persistences are combined with mean chordless cycle densities, and reaches 88.86% when degree-related graph features are added to the training process. The discrepancy between the two models is defined as the absolute value of the difference between their predicted dimensions, computed only for those networks in the validation set on which the two models disagree. The median discrepancy value was found to be 1, with a mean of 1.05 and a standard deviation of 0.2543 when a full vector of descriptors was fed into the model. Additional details are given in Table S3.

When applied to the real-world network dataset described in Tables S4 and S5, the percentage of agreement between the two models was 64.15% over 53 networks with full feature vectors in the models, with a median discrepancy of 1, a mean of 2.95, and a standard deviation of 3.2032, indicating the occurrence of a few larger discrepancies.

In addition, we validated the explanatory power of the predicted dimension by generating surrogate networks from hyperbolic embeddings obtained with *D*-Mercator on real networks. Each network was embedded in the dimension predicted by DIMNN, and the inferred node positions were used to generate surrogate networks. We then compared multiple topological properties to those of the target networks (Figs. S10–S13). Across the tested networks, the surrogates reproduce the degree distribution, clustering spectrum, and _*nn*_ average nearest-neighbor degree, and they typically match algebraic connectivity and average shortest-path length, whereas modularity shows weaker agreement, likely reflecting sensitivity to the chosen community detection method. We also compare bond-percolation curves, finding close agreement in the decay of the giant connected component and the inferred percolation threshold (Fig. S14).

## Discussion

The main contribution of this work is to use densities of cycles (triangles, chordless squares, and chordless pentagons) in a complex network as weights on its edges, and treat each of these three weightings as a filtering function on the underlying graph. Persistence descriptors are computed from the corresponding filtrations and used for estimating the dimensionality of the given network in latent space. The expressiveness of edge-based filtrations defined via densities of chordless cycles is further examined in ref. ^53^, where they are compared with other motif-based filtrations in graph isomorphism detection and property prediction tasks.

Although topological invariants of graphs play a central role in the study of complex networks, methods from topological data analysis have seen limited adoption in this context^1,54^. To the best of our knowledge, extended persistent homology of graphs has not previously been applied to dimensionality estimation in complex networks. Ordinary (as opposed to extended) persistent homology is not well-suited for this purpose, since cycles become permanent in graphs. Using Vietoris–Rips simplicial complexes from graph distances is not suitable in our work either, due to the small-world property, and clique complexes of graphs are not optimal either, since triangles become invisible in clique complexes. The extended persistence or lifetime of a cycle in a weighted graph is the difference between the largest weight and the smallest weight of the edges and nodes that form the given cycle. The total lifetime of a set of linearly independent cycles carries information about the graph's geometric structure.

Other persistence descriptors could be used for analytical purposes. We found that the persistence of connected components along the filtrations was less expressive than its cycle-based counterpart. We also remark that the expressivity of cycle persistence is influenced by certain network parameters, such as inverse temperature (*β*). Indeed, in the point cloud shown in Fig. 2, low values of *β* tend to separate network dimensions less prominently.

Our results show that total extended persistence computed by means of chordless cycle filtrations yields dimensionality estimates which are comparable with those obtained in previous work^44^; see Table 1. Moreover, total persistence improves the accuracy of a neural network classifier if added to feature vectors containing averages of chordless cycle densities and other graph features such as average degree, as shown in Fig. 5 and in Table S2. Our conclusion is that TDA improves dimensionality estimation using a neural network, even with average cycle densities and degree-related graph descriptors.

In fact, estimating latent network dimensionality without generating surrogates is another major contribution in this article. For this purpose, a universal database of synthetic graphs has been generated from a uniformly distributed range of parameter values. Real-world network dimensionality estimates can then be obtained by running a neural network model trained on our database. This approach considerably reduces the computation time of the estimations and the amount of memory required for this task. It is far more ambitious than creating an ensemble of surrogates for each given real network under study, as done in the first part of our work.

Figure 5 highlights the importance of the normalized second moment 〈*k*^2^〉/〈*k*〉^2^ in the accuracy of a neural network classifier for latent dimension. This may be because the normalized second moment correlates with the exponent *γ*, and knowing *β* and *γ* is crucial for dimensionality estimation. An approximation of *β* is provided by the average density of triangles *C*_*t*_, which is also correlated with the total persistence value *TP*_*t*_.

The agreement between two independently trained neural network models, each optimized with a different loss function, is a strong indicator of robustness and generalization. When models trained with distinct objective functions (in our case, cross-entropy for classification and mean squared error for regression) converge to similar predictions on previously unseen data, it is unlikely that their performance is due to overfitting specific patterns or uninformative noise in the training set. Rather, such an agreement suggests that both models are approximating the same underlying relationship between the input features (topological and density-based descriptors) and the latent embedding dimension. It is remarkable that a neural network trained on synthetic networks yields results closely aligned with earlier work on the latent dimension of real-world networks. Moreover, the discrepancies between the dimensionality predictions of the two models may help detect potential out-of-distribution real networks relative to the D-GR model in the study dataset, which therefore deserve further examination.

Our framework primarily targets geometric networks, i.e., those in the regime *β* > *D*. Recent work, however, suggests that some networks may instead lie in a weakly geometric region^55,56^. Extending our approach to this regime and characterizing the interplay between dimensionality and geometricity is a natural direction for future work.

Subsequent research could focus on optimizing the accuracy of a neural network for dimensionality classification of synthetic networks and on improving the performance of latent dimension estimates for real-world networks. On the one hand, this could be achieved by enriching the training database with a wider range of complex network shapes. On the other hand, the neural network architecture could be optimized for the intended tasks. This article only provides a proof of concept for the benefits of a cycle-based filtration in TDA and the feasibility of our deep-learning-assisted dimensionality estimation method. While our architecture (Fig. S5) has demonstrated performance consistent with prior work^44^ and has achieved accuracy above 83% on synthetic networks, improving its overall effectiveness remains a challenge.

A further avenue concerns the distribution shift between the synthetic networks used for training and the real networks used for evaluation. In machine-learning terms, this setting can be viewed as a domain adaptation problem, where a predictor is trained on a source domain (synthetic graphs) and deployed on a related but distinct target domain (real graphs)^57,58^. While our current pipeline does not include an explicit adaptation mechanism, a natural extension would be to augment the model with an adversarial domain discriminator that learns to distinguish synthetic from real networks, while the feature extractor is trained to produce domain-invariant representations, potentially improving transfer and robustness under distribution shift^59^.

## Methods

### Multidimensional geometric soft configuration model

#### The ${{\mathbb{S}}}^{D}/{{\mathbb{H}}}^{D+1}$ model

In the ${{\mathbb{S}}}^{D}$ model^45^, a node *i* is assigned two hidden variables: a hidden degree *κ*_*i*_, quantifying its importance or popularity, and a position **v**_*i*_ in a *D*-dimensional similarity space, represented as a *D*-sphere. The probability of connection between any pair of nodes *i* and *j* follows a gravity law, in which similar nodes are angularly closer and, thus, probably connected^49^. Specifically, nodes *i* and *j* are connected with probability

1 $${p}_{ij}=\frac{1}{1+{\left(\frac{R\Delta {\theta }_{ij}}{{(\mu {\kappa }_{i}{\kappa }_{j})}^{1/D}}\right)}^{\beta }}$$

where *D* is the dimension of the model, *β* controls the level of clustering of the network and the coupling of the network with the underlying metric space, *μ* controls the average degree, and *Δθ*_*ij*_ is the angular distance between nodes *i* and *j*, which are assigned positions **v**_*i*_ and **v**_*j*_ on the *D*-sphere. The radius *R* of the sphere is set such that the density of *N* nodes is 1 (without loss of generality). This yields $R={[\Gamma (\frac{D+1}{2})N/{(2\pi )}^{\frac{D+1}{2}}]}^{1/D}$, where *Γ* is the gamma function^49^. For *β* < *D*, networks are unclustered in the infinite-size limit, whereas for *β* > *D* networks exhibit finite clustering in the thermodynamic limit. Finally, the parameter *μ* controls the average degree of the network and is defined as

2 $$\mu=\frac{\beta \,\Gamma \left(\frac{D}{2}\right)\sin \left(\frac{D\pi }{\beta }\right)}{2{\pi }^{1+\frac{D}{2}}\langle k\rangle }.$$

The ${{\mathbb{S}}}^{D}$ model is isomorphic to the purely geometric ${{\mathbb{H}}}^{D+1}$ model^46^ in (*D* + 1)-hyperbolic space by mapping the hidden degree into the radial coordinates as

3 $${r}_{i}=\widehat{R}-\frac{2}{D}{\mathrm{ln}}\frac{{\kappa }_{i}}{{\kappa }_{0}},\,\,\mathrm{with}\,\,\widehat{R}=2\,{\mathrm{ln}}\left(\frac{2R}{{(\mu {\kappa }_{0}^{2})}^{1/D}}\right).$$

A network generation procedure following the ${{\mathbb{S}}}^{D}/{{\mathbb{H}}}^{D+1}$ model is described in Algorithm 1 in Supplementary Information, where a cutoff *κ*_*c*_ is calculated to prevent excessive fluctuations in the largest expected degrees when *γ* < 3; see ref. ^60^.

#### Microcanonical formulation of ${{\mathbb{S}}}^{D}$ model

A microcanonical version of the ${{\mathbb{S}}}^{1}$ model was first proposed in ref. ^51^, where it was called geometric randomization model (GR). The GR operates on the sequence of observed node degrees and assigns node positions at random in the similarity space. The network is rewired to maximize the likelihood that the new topology is generated by the ${{\mathbb{S}}}^{1}$ model while preserving the observed degrees and, thus, the total number of edges.

In this work, we extended GR to higher-dimensional similarity spaces, which we call D-GR. We used D-GR to generate synthetic networks for dimensionality estimation when investigating the relationship between total persistence in homological dimension 1 computed from three types of chordless cycles filtrations and the inferred dimension (see Section 2.1). We want to highlight the main difference with respect to the previous approach^44^. In ref. ^44^, the authors infer the set of degrees *κ* from a given network and generate synthetic networks with Eq. (1) given the parameters *β* and *D*. Although in ref. ^44^ the degree distribution of the generated surrogates is very similar to that of the input network, the D-GR procedure is more constrained and maintains the exact degree values of the input network.

In the D-GR model, we assign to each node *i* a random position in the *D* + 1 dimensional Euclidean space ${{{\bf{v}}}}_{i}\in {{\mathbb{R}}}^{D+1}$ with ∣∣**v**_*i*_∣∣ = *R*. The nodes are uniformly distributed on the *D*-sphere using Marsaglia’s algorithm^61^. The rewiring procedure is carried out with the Metropolis–Hastings algorithm, aimed at finding the adjacency matrix that maximizes the likelihood function

4 $${{\mathcal{L}}}={\prod }_{i < j}{p}_{ij}^{{a}_{ij}}{[1-{p}_{ij}]}^{1-{a}_{ij}}$$

where *p*_*ij*_ comes from Eq. (1) and *a*_*ij*_ are elements of the adjacency matrix. The method proceeds as described in Algorithm 2 in Supplementary Information.

As shown in ref. ^51^, the probability of swapping links between nodes *i* and *j* and between nodes *l* and *m* is given by

5 $$\frac{{{{\mathcal{L}}}}_{n}}{{{{\mathcal{L}}}}_{c}}={\left(\frac{\Delta {\theta }_{ij}\Delta {\theta }_{lm}}{\Delta {\theta }_{il}\Delta {\theta }_{jm}}\right)}^{\beta }.$$

Notice that Eq. (5) does not depend on the dimension *D*.

To validate our approach, we generated synthetic networks with known dimension using the ${{\mathbb{S}}}^{D}$ model (see Algorithm 1 in the Supplementary Information), with the following parameters: the number of nodes (*N*) was set to 500 and the average degree 〈*k*〉 to 10; dimension (*D*) ranging from 1 to 7; the inverse temperature parameter (*β*) taking values 1.5 *D* and 2.5 *D*; and the power-law exponent (*γ*) taking values 2.7 and 3.5. For each set of parameters, we generated 10 network realizations, thus obtaining 280 synthetic networks. For each of these, we produced a set of surrogates using the D-GR method (Algorithm 2 in the Supplementary Information), scanning over different values: dimension (*D*) ranging from 1 to 7 and rescaled inverse temperature *β*/*D* ranging from 1.2 to 3.0 with steps of 0.1. The geometric randomization was repeated 10 times, yielding 1 330 surrogates per synthetic network. At the end of this procedure, we obtained a total of 372 400 networks, that were used in the confusion matrices in Fig. 3.

To infer the dimension of real networks, we followed a similar approach. We applied the D-GR method with the same set of parameters described above, yielding a total of 1 330 surrogates for each real network. Inferred dimensions of real networks are described in Table 1.

It is worth noting some limitations of this method. The acceptance probability depends on the parameter *β*. For very large values of *β*, the acceptance ratio becomes binary, i.e., moves that increase the likelihood are almost always accepted, and those that decrease it are almost always rejected, and the likelihood plateau cannot be reached. Thus, the algorithm is restricted to moderate values of *β* and the corresponding clustering coefficient. Techniques such as simulated annealing or parallel tempering can restore good mixing in the large *β* regime. In this work, however, we restrict our attention to real networks whose topology is well-captured by a moderate value of *β*.

Moreover, even though the ${{\mathbb{S}}}^{D}/{{\mathbb{H}}}^{D+1}$ model captures a wide range of topological network properties, some real networks may lie outside the range of values and are located further away from the surrogate networks in the persistence (*TP*_*t*_, *TP*_*s*_, *TP*_*p*_) and mean cycle density (*C*_*t*_, *C*_*s*_, *C*_*p*_) configuration spaces.

### Graph features

#### Densities of chordless cycles

Let *G* = (*V*, *E*) be a graph and *e*_*ij*_ = {*v*_*i*_, *v*_*j*_} be an edge between nodes *v*_*i*_ and *v*_*j*_ in *E*, with degrees *k*_*i*_ > 1 and *k*_*j*_ > 1, respectively. The density of triangles corresponding to the edge *e*_*ij*_, also called edge clustering coefficient^62^, is the number *#*Δ_*ij*_ of edge triangles in *G* containing *e*_*ij*_ divided by the maximum possible number of triangles in *G* containing *e*_*ij*_ given the degrees *k*_*i*_ and *k*_*j*_, that is,

6 $${C}_{t}({e}_{ij})=\frac{\#{\Delta }_{ij}}{\min ({k}_{i},{k}_{j})-1}.$$

An edge cycle is chordless if there is no edge between its nodes except those that form the cycle. Density of squares, denoted *C*_*s*_(*e*_*ij*_), is defined by dividing the number of chordless edge squares in *G* containing *e*_*ij*_ by the maximum possible number of such squares given the degrees *k*_*i*_ and *k*_*j*_ and the existing triangles through *e*_*ij*_ in *G*. To define the density of pentagons *C*_*p*_(*e*_*ij*_) per edge *e*_*ij*_, we count the number of chordless pentagons containing *e*_*ij*_ and normalize it by the maximum possible number of such pentagons, assuming the degrees of *v*_*i*_ and *v*_*j*_ and the degrees of their respective neighbors. One could also define similar densities per node. However, in ref. ^62^, the authors found the definitions relative to edges to be more stable with respect to degree heterogeneity than those relative to nodes.

Regarding the computational cost of computing chordless cycles, the time complexities for computing densities of triangles, squares, and pentagons are *O*(*N*〈*k*〉^2^), *O*(*N*〈*k*〉^3^), and *O*(*N*〈*k*〉^4^), respectively, where 〈*k*〉 is the average degree and *N* = *#E* is the number of edges. In sparse graphs, 〈*k*〉 ≪ *N*. Although the computational cost for large graphs may be high, the computations can be easily parallelized or implemented on a GPU using CUDA^63^.

#### Extended persistent homology

In this work, we use persistent homology to extract information from a graph *G* equipped with a filtration {*G*_*t*_}, where *t* is a real-valued parameter. Persistence refers to the evolution of cycles along the values of the given filtration.

Homology of graphs is a special case of simplicial homology of simplicial complexes^64^. In the case of graphs, homology is determined by two numbers, called Betti numbers, namely the number *β*_0_ of connected components and the cardinality *β*_1_ of a maximal set of linearly independent cycles. Cycles are finite formal sums *z* = ∑*λ*_*i*_*e*_*i*_ of oriented edges with coefficients *λ*_*i*_ = ± 1 such that ∑*λ*_*i*_∂*e*_*i*_ = 0, where, for an edge *e* ∈ *E* from *v*_0_ to *v*_1_, we denote ∂*e* = *v*_1_ − *v*_0_. Thus, cycles are algebraic representations of closed edge paths in the given graph.

In a filtered simplicial complex {*K*_*t*_}, the birth of a cycle *z* of any dimension is the *t*-value at which *z* appears in *K*_*t*_, and the death of *z* is the *t*-value at which *z* becomes the boundary of a higher chain. Hence, in the case of a filtered graph, the birth of a cycle *z* is the *t*-value at which *z* is formed, and the death value is infinite, since a graph does not contain higher simplices.

To avoid infinite persistence values, we use extended persistence, as in refs. ^65,66^. For a graph *G* = (*V*, *E*) equipped with a node weighting ${w}_{V}:V\to {\mathbb{R}}$, the sublevel filtration {*G*_*t*_} is defined as *G*_*t*_ = (*V*_*t*_, *E*_*t*_), where *V*_*t*_ = {*v* ∈ *V *∣ *w*_*V*_(*v*) ≤ *t*} and *E*_*t*_ is the subset of *E* spanned by *V*_*t*_, and the superlevel filtration {*G*^*t*^} is defined as *G*^*t*^ = (*V*^*t*^, *E*^*t*^), where *V*^*t*^ = {*v* ∈ *V *∣ *w*_*V*_(*v*) ≥ *t*} and *E*^*t*^ is the subset of *E* spanned by *V*^*t*^. Then the extended persistence of a cycle *z* in *G* is defined as ∣*d* − *b*∣, where *b* is the birth value of *z* in the sublevel filtration {*G*_*t*_} and *d* is the birth value of *z* in the superlevel filtration {*G*^*t*^}. Note, however, that no edge weighting can be defined on *G* compatibly with *w*_*V*_, which is consistent with both {*G*_*t*_} and {*G*^*t*^}, in general.

In our work, chordless cycle filtrations are defined by means of edge weightings, not node weightings. Although it is perfectly possible to exchange the roles of nodes and edges in the definitions of sublevel and superlevel filtrations, extended persistence calculations have been carried out using the Python library Gudhi^67^, which only provides software for computing extended persistence of graphs with a weighting on their nodes (see Section 2.1 of ref. ^66^). Therefore, we introduce a method to replace a graph *G* = (*V*, *E*) equipped with an edge weighting ${w}_{E}:E\to {\mathbb{R}}$ by a topologically equivalent graph ${G}^{{\prime} }=({V}^{{\prime} },{E}^{{\prime} })$ in which $E\subseteq {E}^{{\prime} }$ and endowed with a node weighting ${w}_{{V}^{{\prime} }}:{V}^{{\prime} }\to {\mathbb{R}}$ yielding the same extended persistence diagram as *G*.

To achieve this, we split each node *v*_*i*_ ∈ *V* of degree *k*_*i*_ into *k*_*i*_ distinct nodes ${v}_{i,j}\in {V}^{{\prime} }$, where *j* ranges over the subindices of the neighbors of *v*_*i*_. Each node *v*_*i*,*j*_ is assigned weight ${w}_{{V}^{{\prime} }}({v}_{i,j})={w}_{E}({e}_{ij})$ where *e*_*ij*_ = {*v*_*i*_, *v*_*j*_}. The set ${E}^{{\prime} }$ contains horizontal edges {*v*_*i*,*j*_, *v*_*j*,*i*_} for all *i*, *j*, joining nodes with the same weight, and vertical edges, connecting each string of nodes *v*_*i*,*j*_ with a fixed *i* value, sequentially from smallest weight to largest, as in Fig. 1. Hence, the set of horizontal edges in ${E}^{{\prime} }$ is in bijective correspondence with *E*, and the weighting ${w}_{{V}^{{\prime} }}$ is consistent with *w*_*E*_ on all edges of *E*, in both the sublevel and superlevel filtrations of ${G}^{{\prime} }$.

We call ${G}^{{\prime} }$ a degree-splitting subdivision of *G*. An example is shown in Fig. 1, and a corresponding sequence of sublevel graphs and superlevel graphs explaining the choice of generating cycles for extended persistence is depicted in Fig. S4.

The graph ${G}^{{\prime} }$ is then fed into Gudhi^67^ as a node-weighted graph, and total persistence ∑∣*d*_*i*_ − *b*_*i*_∣ of cycles in the corresponding extended persistence diagram is recorded (relative cycles provided by the Gudhi software, if any, are discarded). The graph ${G}^{{\prime} }$ has the same Betti numbers as *G* at the same thresholds, since *G* is obtained from ${G}^{{\prime} }$ by contracting vertical edges, which does not change homology.

#### Degree-related graph features

The following descriptors from complex network theory are calculated to train the models used in Section 2.2. For each graph *G* = (*V*, *E*), we consider the average degree of its nodes, 〈*k*〉 = (1/*#V*)∑_*v*∈*V *_deg(*v*). The minimal degree ${k}_{\min }$ and maximal degree ${k}_{\max }$ are also considered. The average neighbor degree of *G* is the average of the mean neighbor degree of its nodes:

$$\langle {k}_{\rm nn}\rangle=\frac{1}{\#V}\mathop{\sum }\limits_{v\in V}\left(\frac{1}{{\rm{deg}}(v)}\mathop{\sum }\limits_{u \sim v}{\rm{deg}}(u)\right).$$

The normalized second moment of *G* is defined as the quotient 〈*k*^2^〉/〈*k*〉^2^, where we denote 〈*k*^2^〉 = (1/*#V*)∑_*v*∈*V *_deg(*v*)^2^. Its significance is due to the fact that it correlates inversely with the power-law exponent *γ* in scale-free networks^68^.

### Database and neural network architecture

For better understanding and comparison of the results, we used the same real-world networks as in ref. ^44^. These are undirected networks with fewer than 100,000 nodes from very different domains. Tables S4 and S5 include a detailed description of the data as reported in ref. ^44^.

#### SYNNET dataset for inferring dimension using neural networks

Deep networks require large amounts of data to avoid overfitting and to learn robust features, due to their large number of parameters. In fact, neural networks generally require large datasets to achieve high performance^69,70^. However, with the ${{\mathbb{S}}}^{D}$ model, we tackle this challenge by generating plenty of synthetic graphs with known dimensions to train neural networks.

We prepared a dataset of 792 000 synthetic networks generated using the ${{\mathbb{S}}}^{D}$ model by means of the method described in Algorithm 1 in the Supplementary Information with the following input parameters: dimension (*D*) from 1 to 10; number of nodes (*N*) with values 200, 400, 750, 1000, 2500; power-law exponent (*γ*) with values 2.2, 2.4, 3.0, 4.0, 5.0; average degree (〈*k*〉) with values 4, 8, 12, 25; and rescaled inverse temperature (*β*/*D*) with values 1.2, 1.4, 1.6, 1.8, 2.0, 2.2, 2.5, 2.8, 3.0, 3.5, 4.0, 5.0. For each set of parameters, 66 network realizations were made. The dataset covers a wide range of network properties —in particular, 〈*k*_*nn*_〉 ∈ [4.4, 300.8], *C*_*t*_ ∈ [0.073, 0.891], *C*_*s*_ ∈ [0.00505, 0.24193], *C*_*p*_ = [0.00010, 0.02165].

The database contains a set of feature vectors for each network, aiming for its use in a multilayer perceptron (MLP), which is described in Section 4.3. The following descriptors were computed, resulting in a 12-dimensional feature vector: *N*, 〈*k*〉, 〈*k*^2^〉/〈*k*〉^2^, ${k}_{\min }$, ${k}_{\max }$, 〈*k*_nn_〉, *C*_*t*_, *C*_*s*_, *C*_*p*_, *TP*_*t*_, *TP*_*s*_, *TP*_*p*_, as described in Section 4.2. Once a MLP is trained, the same features are computed for real networks in order to predict their dimensions.

It is worth noting that some real networks may exhibit topological properties not included in our dataset. Hence, neural networks might be prone to misclassifying them. We can overcome this issue by expanding the range or parameter values used to generate the dataset. In the released code, we provide a check for the out-of-distribution parameters.

#### Neural network DIMNN model

For the classification task, a deep multilayer perceptron (MLP) architecture combined with residual network (ResNet) techniques was used, as in ref. ^71^. Deeper neural networks are capable of capturing highly non-linear relationships in data, but training very deep architectures often leads to optimization difficulties such as vanishing gradients. ResNets address these issues by introducing skip (residual) connections, which add the input of a layer to its output. Such mappings facilitate gradient flow and stabilize training, enabling the construction of deeper and more expressive models^72^.

The proposed architecture integrates residual connections into a fully connected MLP, enabling the model to benefit from deep representation while maintaining stable training dynamics. The network comprises 21 hidden layers with ReLu activations, and the configuration depicted in Fig. S5. The output layer uses a softmax activation. A dropout regularization of 50% is applied throughout the network to mitigate overfitting and enhance generalization. The training process was carried out using AdamW as optimizer, with a learning rate of 0.0005 and incorporating early stopping to prevent overfitting. This architecture benefits from the expressive power of deep learning while leveraging the robustness of residual learning, making it well-suited to the proposed classification task.

## Supplementary information

Supplementary Information

Transparent Peer Review file

## Data Availability

The real network datasets used in this study are available from the sources referenced in the manuscript and the Supplementary Information. The SYNNET dataset of 792 000 synthetic networks generated with the ${{\mathbb{S}}}^{D}$ model used for neural network training, along with the neural network checkpoints, is available on Zenodo^73^.

## Code availability

The open-source code generated in this work, along with the code to reproduce the figures, is available on GitHub at https://github.com/networkgeometry/detecting-dimensionality-TDA-DimNN.
