# Supplementary material for: Chordless cycle filtrations for dimensionality detection in complex networks via topological data analysis
Source: Nat Commun. 2026 May 6;17:6105. doi: 10.1038/s41467-026-72687-z (PMC13358065; doi:10.1038/s41467-026-72687-z)
Supplement: Supplementary file 1 — Supplementary Information [file 41467_2026_72687_MOESM1_ESM.pdf]

Supplementary Information for "Chordless cycle filtrations for dimensionality detection in complex networks via topological data analysis"

Aina Ferrà Marcús,[1] Robert Jankowski,[2,3,4] Meritxell Vila-Miñana,[5] Carles Casacuberta,[1] and M. Ángeles Serrano[2,3,6,*]

[1] *Departament de Matèmatiques i Informàtica, Universitat de Barcelona, Gran Via de les Corts Catalanes 585, 08007 Barcelona, Spain*
[2] *Departament de Física de la Matèria Condensada, Universitat de Barcelona, Martí i Franquès 1, E-08028 Barcelona, Spain*
[3] *Universitat de Barcelona Institute of Complex Systems (UBICS), Universitat de Barcelona, Barcelona, Spain*
[4] *Faculty of Electrical Engineering, Mathematics and Computer Science, Delft University of Technology, 2628 CD, Delft, Netherlands*
[5] *Center for Complex Networks and Systems Research, Luddy School of Informatics, Computing, and Engineering, Indiana University, Bloomington, IN, USA*
[6] *ICREA, Passeig Lluís Companys 23, E-08010 Barcelona, Spain*

CONTENTS

1. Dimensionality estimation of real networks with persistence descriptors 2
2. Neural network architecture and predictions 6
3. Topological properties of real networks and their inferred dimensions 10
4. Network generation algorithms 14
5. Validation with D-Mercator 15

Supplementary References 17

* marian.serrano@ub.edu

1. DIMENSIONALITY ESTIMATION OF REAL NETWORKS WITH PERSISTENCE DESCRIPTORS

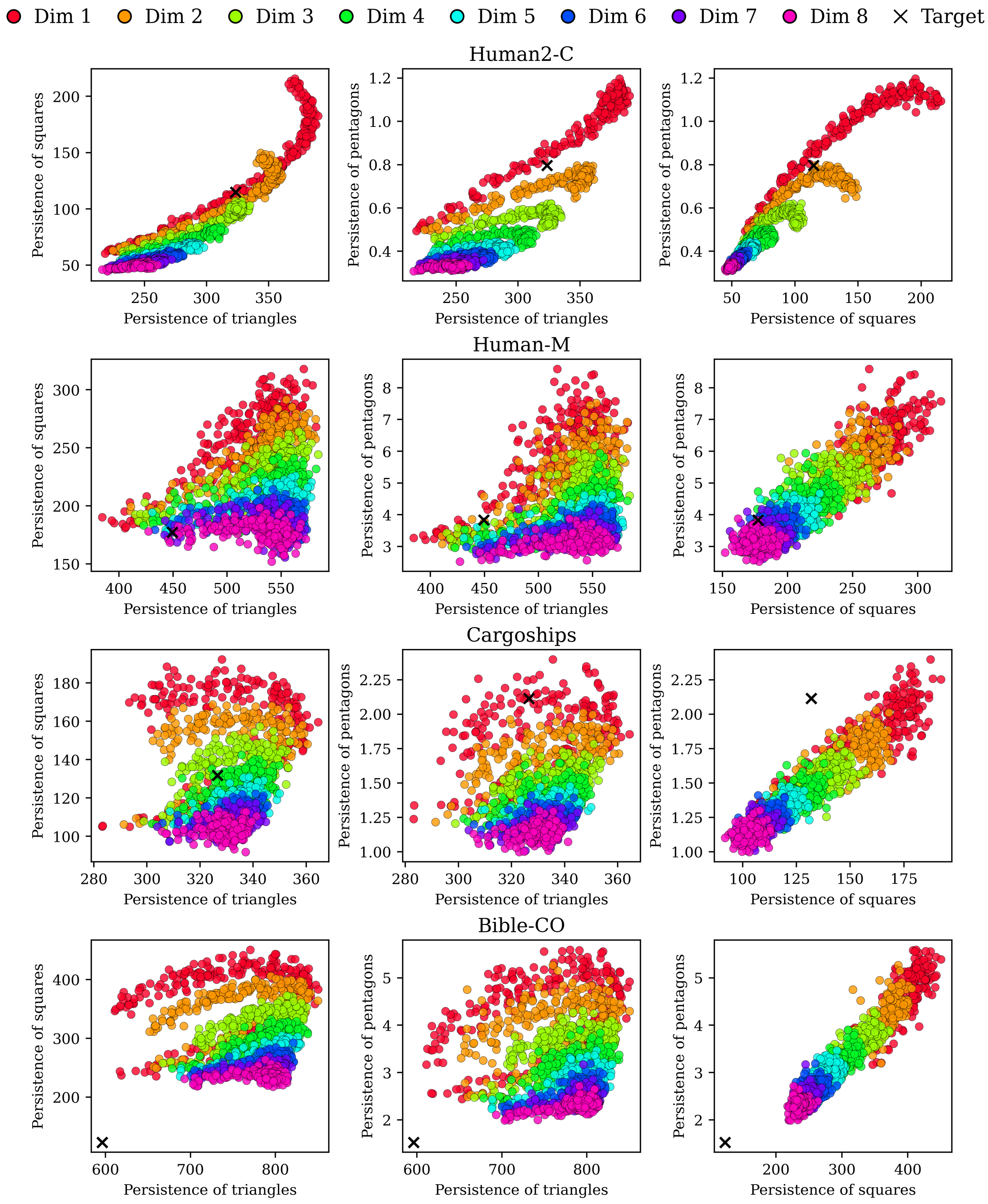

FIG. S1: 2D projections representing an ensemble of 1330 surrogates for each real network in the phase space of total persistence computed from three chordless cycle densities (triangles, squares, and pentagons). Points are colored by dimension, and the target network is marked with a black cross. Part 1 of 2.

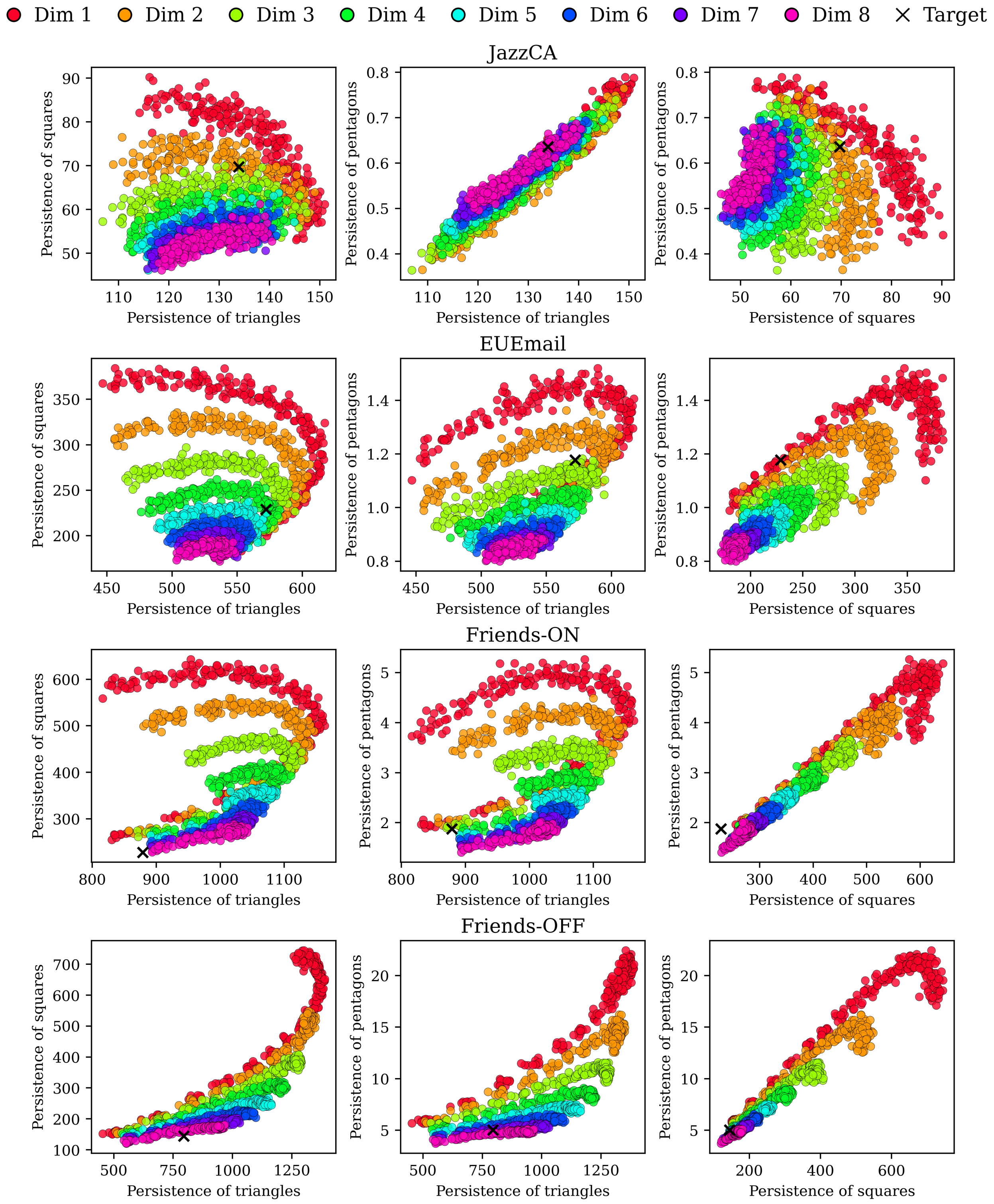

FIG. S2: 2D projections representing an ensemble of 1330 surrogates for each real network in the phase space of total persistence computed from three chordless cycle densities (triangles, squares, and pentagons). Points are colored by dimension, and the target network is marked with a black cross. Part 2 of 2.

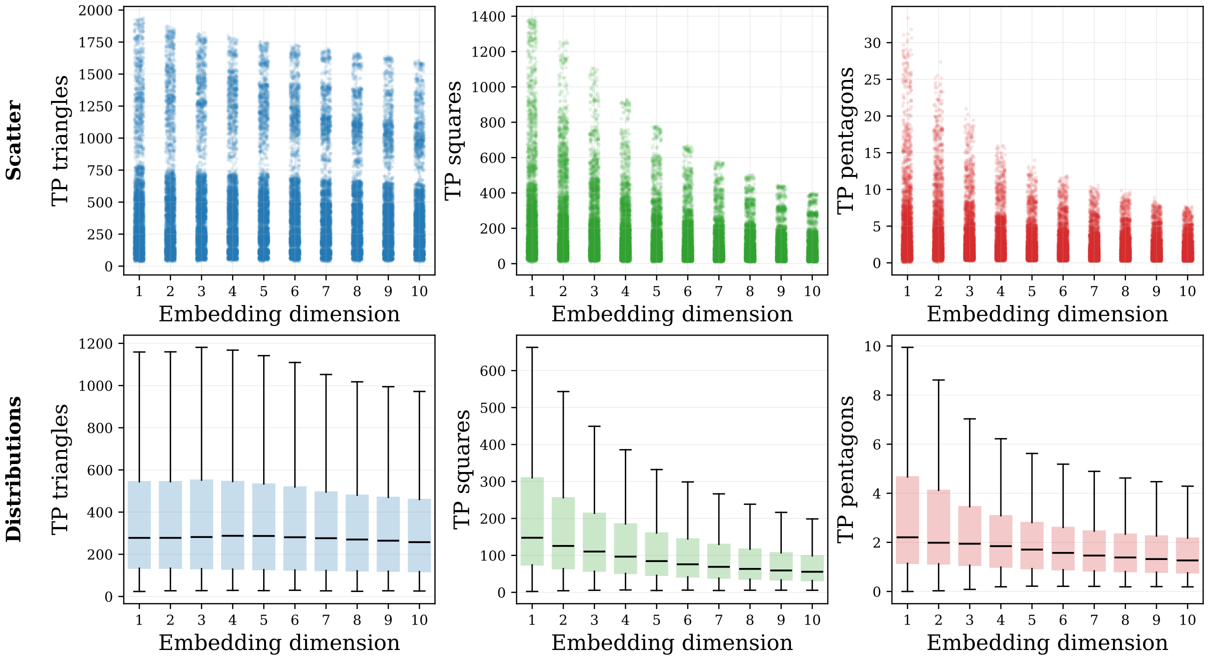

FIG. S3: Inverse relationship between network dimension (x-axis) and total extended persistence of cycles (y-axis) for the three filtrations considered: triangles (left), squares (middle), and pentagons (right). *Top row:* Jittered scatter plots; *Bottom row:* Boxplots summarizing the distribution within each dimension. These distributions have been obtained from a sample of 50 000 graphs from the SynNet database, uniformly distributed with respect to dimension.

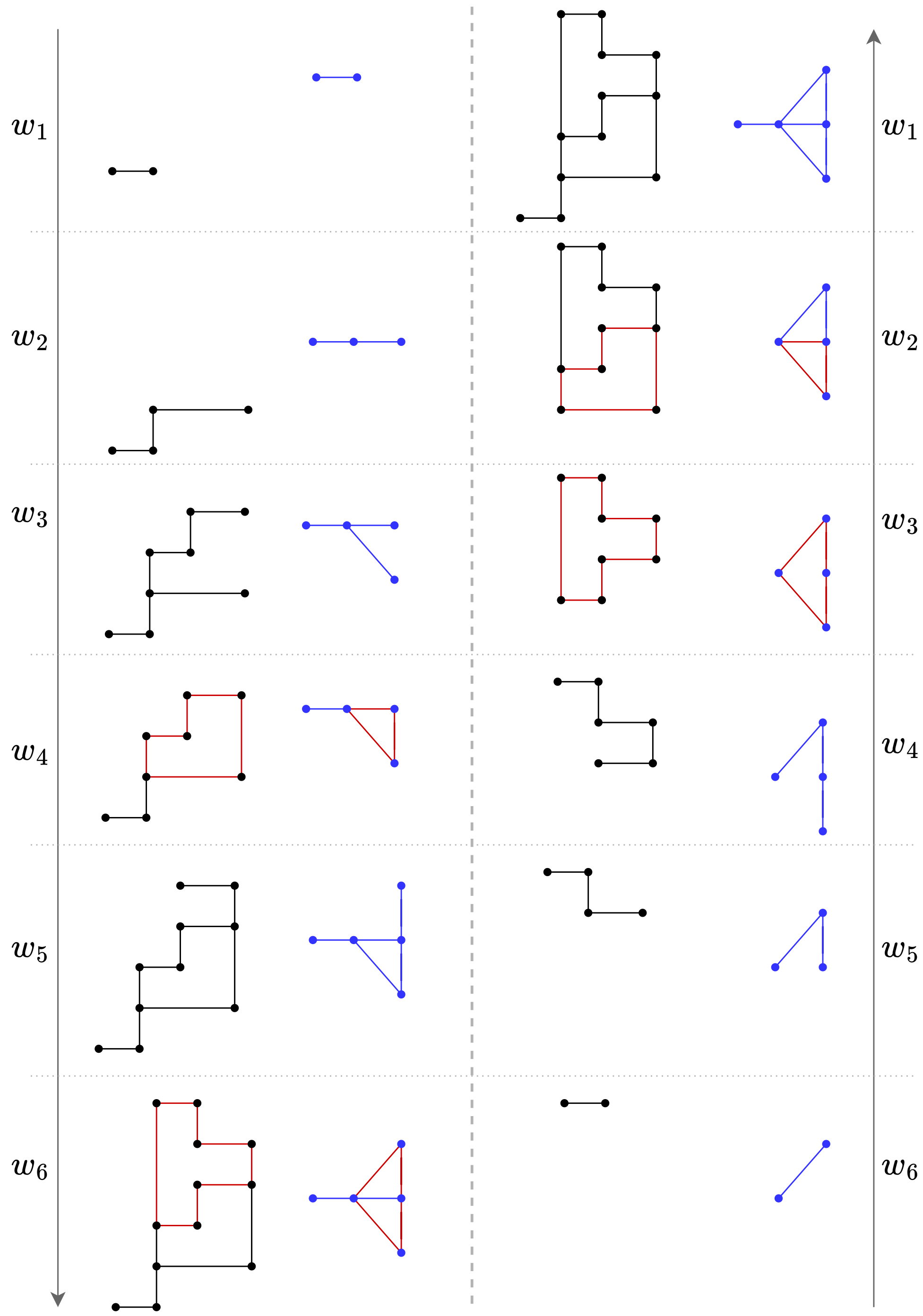

FIG. S4: Creation and disappearance of cycles along the sublevel graph filtration (left) followed by the superlevel graph filtration (right) on the example graphs G and G' shown in Fig. 1. The first generating cycle is born at w_4 and dies at w_2 (i.e., it appears at w_2 in the superlevel graph filtration), while the second generating cycle is born at w_6 and dies at w_3. *Blue:* Original edge-weighted graph G; *Black:* Node-weighted graph G' obtained from G by degree-splitting subdivision.

2. NEURAL NETWORK ARCHITECTURE AND PREDICTIONS

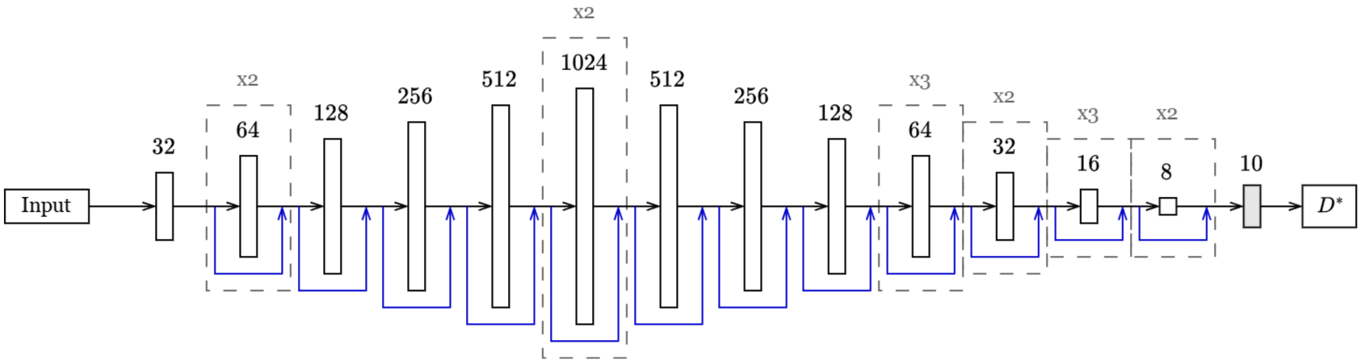

FIG. S5: Schematic representation of the DimNN neural network architecture. Boxes indicate layers within the MLP, with the number above each box specifying the number of neurons in that layer. Blue arrows are used to denote skip (residual) connections. Dashed gray boxes denote repeated layer blocks, with the repetition count indicated above each block. The output layer is shaded in gray and employs a softmax activation, yielding a 10-dimensional output corresponding to the number of classes in the classification problem.

TABLE S1: Ranges of the feature values present in the SynNet training set. Feature vectors falling outside these intervals are out of distribution and may lead to misclassifications.

**Feature**	**Mean**	**Std**	**Min**	**Max**
N	970.00	813.39	200.00	2500.00
$\langle k \rangle$	12.25	7.89	4.00	25.00
$\langle k^2 \rangle / \langle k \rangle^2$	2.03	1.26	1.04	13.09
$k_{\min}$	2.63	2.85	1.0	16.00
$k_{\max}$	125.65	165.87	8.0	1734.00
$\langle k_{\text{nn}} \rangle$	26.47	24.80	4.38	300.86
C_t	0.41	0.16	0.07	0.89
C_s	0.07	0.03	0.01	0.24
C_p	0.0019	0.0013	0.0000	0.0216
TP_t	416.77	387.48	23.76	1941.44
TP_s	136.87	158.95	2.40	1394.54
TP_p	2.43	2.67	0.00	34.75

TABLE S2: Validation accuracies after five repetitions of DIMNN, a ResNet training with AdamW optimizer using the SYNNET network database. Columns correspond to features included in each model: N number of nodes; $\langle k \rangle$ average degree; $\langle k^2 \rangle / \langle k \rangle^2$ normalized second moment; k_{min} minimum degree; k_{max} maximum degree; $\langle k_{\mathrm{nn}} \rangle$ mean average neighbor degree; C_t average triangle density; C_s average chordless square density; C_p average chordless pentagon density; TP_t, TP_s, TP_p total persistence in homological dimension 1 computed from chordless cycle filtrations (triangles, squares, and pentagons, respectively).

N	$\langle k \rangle$	$\langle k^2 \rangle / \langle k \rangle^2$	k_{min}	k_{max}	$\langle k_{\mathrm{nn}} \rangle$	C_t	C_s	C_p	TP_t	TP_s	TP_p	Accuracy (%)	Time (sec)	Epochs
						✓	✓	✓				53.20 ± 2.15	7529.71	73.40
✓						✓	✓	✓				66.79 ± 0.67	7582.86	73.60
✓	✓					✓	✓	✓				68.95 ± 2.08	5718.05	55.50
✓	✓	✓				✓	✓	✓				81.53 ± 0.67	6372.85	61.40
✓	✓	✓	✓	✓		✓	✓	✓				80.96 ± 0.46	5449.96	52.75
✓	✓	✓	✓	✓	✓	✓	✓	✓				82.32 ± 1.39	4890.98	47.40
									✓	✓	✓	56.06 ± 1.17	8326.58	74.40
✓									✓	✓	✓	59.02 ± 2.73	6348.86	65.00
✓	✓								✓	✓	✓	59.45 ± 1.52	6015.84	60.00
✓	✓	✓							✓	✓	✓	71.60 ± 2.30	6563.73	64.67
✓	✓	✓	✓	✓					✓	✓	✓	70.74 ± 2.57	6663.77	65.80
✓	✓	✓	✓	✓	✓				✓	✓	✓	73.10 ± 1.19	5592.25	54.80
						✓	✓	✓	✓	✓	✓	72.64 ± 3.32	6518.76	59.20
✓						✓	✓	✓	✓	✓	✓	74.57 ± 1.86	6343.14	57.00
✓	✓					✓	✓	✓	✓	✓	✓	74.39 ± 2.76	7928.53	59.40
✓	✓	✓				✓	✓	✓	✓	✓	✓	81.83 ± 0.44	7682.31	53.00
✓	✓	✓	✓	✓		✓	✓	✓	✓	✓	✓	81.41 ± 0.38	5778.89	37.60
✓	✓	✓	✓	✓	✓	✓	✓	✓	✓	✓	✓	**83.00 ± 0.38**	4796.51	42.60

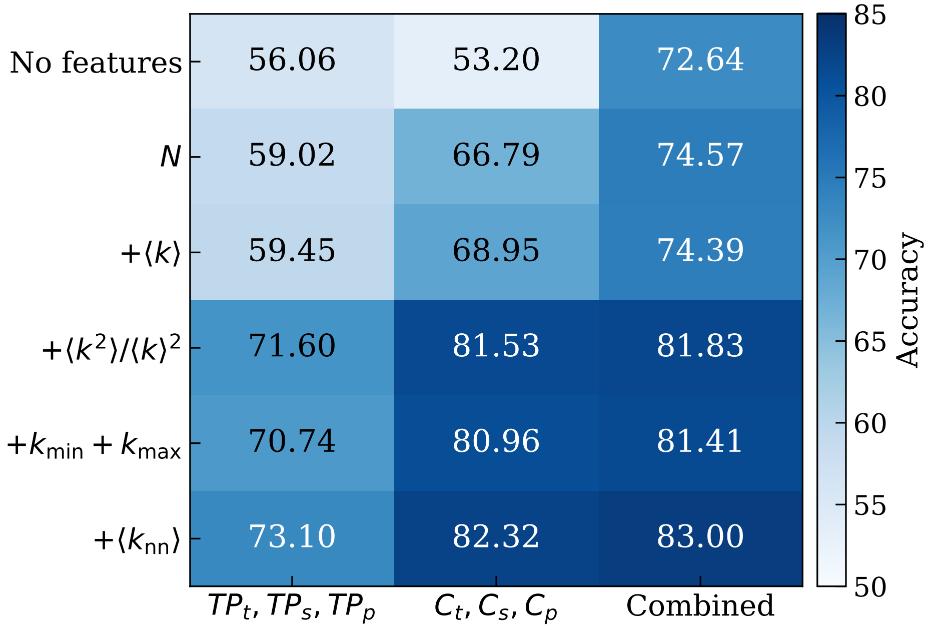

FIG. S6: Impact of cycle density and TDA features on DIMNN validation accuracy on the SYNNET dataset. Heatmap of mean validation accuracy for five progressively enriched input network features. The results are averaged over five neural network initializations.

TABLE S3: Comparative measures for each combination of graph features between the predictions of the DimNN classifier and a regressor with the same architecture and mean squared error as loss function, averaged over five repetitions. Discrepancy is computed from the collection of SynNet networks in the validation set on which the two models do not agree. *Agreement:* Percentage of agreement between the two models; *Median:* Median discrepancy; *Q3:* Third quartile of discrepancy; *Mean:* Mean of discrepancy; *Std:* Standard deviation of discrepancy.

**Descriptors**	**Agreement (%)**	**Median**	**Q3**	**Mean**	**Std**
C_t, C_s, C_p	44.31	1.0	2.0	1.41	0.69
N, C_t, C_s, C_p	67.12	1.0	1.0	1.19	0.46
$N, \langle k \rangle, C_t, C_s, C_p$	73.18	1.0	1.0	1.18	0.45
$N, \langle k \rangle, \langle k^2 \rangle / \langle k \rangle^2, C_t, C_s, C_p$	88.45	1.0	1.0	1.04	0.24
$N, \langle k \rangle, \langle k^2 \rangle / \langle k \rangle^2, k_{\min}, k_{\max}, C_t, C_s, C_p$	88.19	1.0	1.0	1.06	0.30
$N, \langle k \rangle, \langle k^2 \rangle / \langle k \rangle^2, k_{\min}, k_{\max}, \langle k_{\mathrm{nn}} \rangle, C_t, C_s, C_p$	88.90	1.0	1.0	1.05	0.27
TP_t, TP_s, TP_p	58.83	1.0	1.4	1.30	0.63
N, TP_t, TP_s, TP_p	67.75	1.0	1.0	1.22	0.56
$N, \langle k \rangle, TP_t, TP_s, TP_p$	71.06	1.0	1.0	1.18	0.48
$N, \langle k \rangle, \langle k^2 \rangle / \langle k \rangle^2, TP_t, TP_s, TP_p$	79.14	1.0	1.0	1.12	0.39
$N, \langle k \rangle, \langle k^2 \rangle / \langle k \rangle^2, k_{\min}, k_{\max}, TP_t, TP_s, TP_p$	77.70	1.0	1.0	1.12	0.40
$N, \langle k \rangle, \langle k^2 \rangle / \langle k \rangle^2, k_{\min}, k_{\max}, \langle k_{\mathrm{nn}} \rangle, TP_t, TP_s, TP_p$	80.43	1.0	1.0	1.11	0.40
$C_t, C_s, C_p, TP_t, TP_s, TP_p$	79.33	1.0	1.0	1.05	0.24
$N, C_t, C_s, C_p, TP_t, TP_s, TP_p$	79.62	1.0	1.0	1.06	0.27
$N, \langle k \rangle, C_t, C_s, C_p, TP_t, TP_s, TP_p$	78.65	1.0	1.0	1.05	0.24
$N, \langle k \rangle, \langle k^2 \rangle / \langle k \rangle^2, C_t, C_s, C_p, TP_t, TP_s, TP_p$	88.38	1.0	1.0	1.04	0.24
$N, \langle k \rangle, \langle k^2 \rangle / \langle k \rangle^2, k_{\min}, k_{\max}, C_t, C_s, C_p, TP_t, TP_s, TP_p$	87.79	1.0	1.0	1.06	0.28
$N, \langle k \rangle, \langle k^2 \rangle / \langle k \rangle^2, k_{\min}, k_{\max}, \langle k_{\mathrm{nn}} \rangle, C_t, C_s, C_p, TP_t, TP_s, TP_p$	88.86	1.0	1.0	1.05	0.25

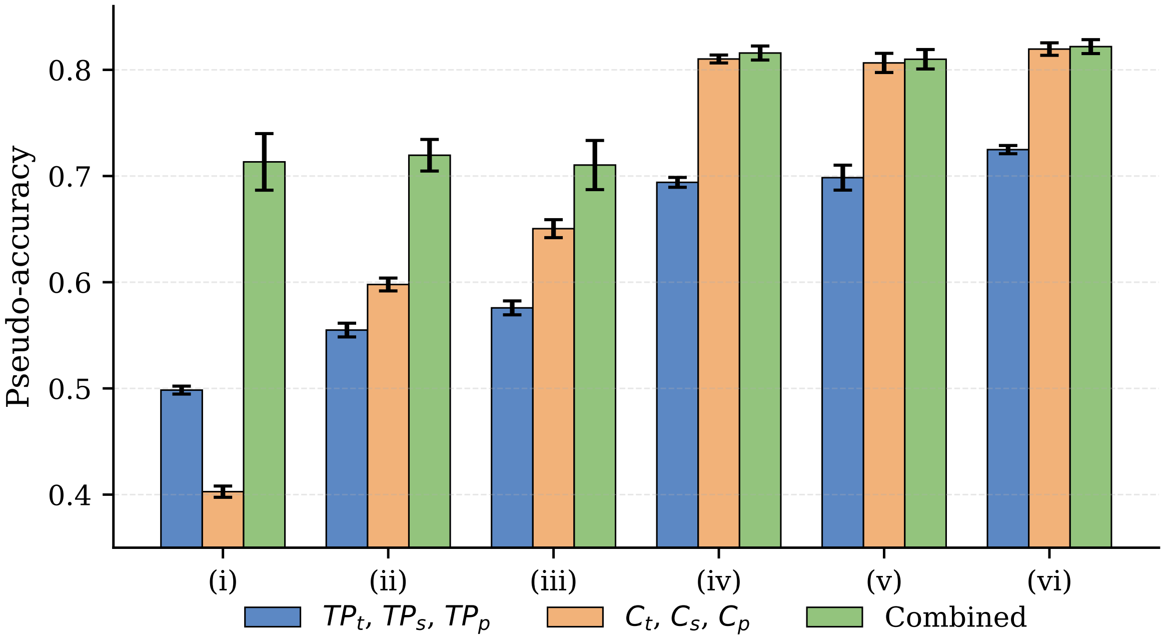

FIG. S7: Averages and standard deviations of pseudo-accuracy on a validation set after five runs of a neural network regressor using the SynNet database. Pseudo-accuracy refers to the fact that the prediction of a regressor is a real number, which is approximated to the nearest integer. *Blue:* Accuracies obtained using total persistences TP_t, TP_s, TP_p, plus cumulative features; *Orange:* Accuracies using average cycle densities C_t, C_s, C_p, plus cumulative features; *Green:* Accuracies obtained by combining total persistences and mean cycle densities plus cumulative features. Successive columns correspond to incorporating one after the other the following additional features into the model: (i) no added features; (ii) number of nodes N; (iii) number of nodes and average degree $\langle k \rangle$; (iv) number of nodes, average degree, and normalized second moment $\langle k^2 \rangle / \langle k \rangle^2$; (v) number of nodes, average degree, normalized second moment, minimum degree $k_{\min}$ and maximum degree $k_{\max}$; (vi) number of nodes, average degree, normalized second moment, minimum degree, maximum degree, and mean average neighbor degree $\langle k_{\rm nn} \rangle$.

3. TOPOLOGICAL PROPERTIES OF REAL NETWORKS AND THEIR INFERRED DIMENSIONS

We compile a dataset of 53 real-world networks from various domains. For more details about each network, we refer to [1] and [2].

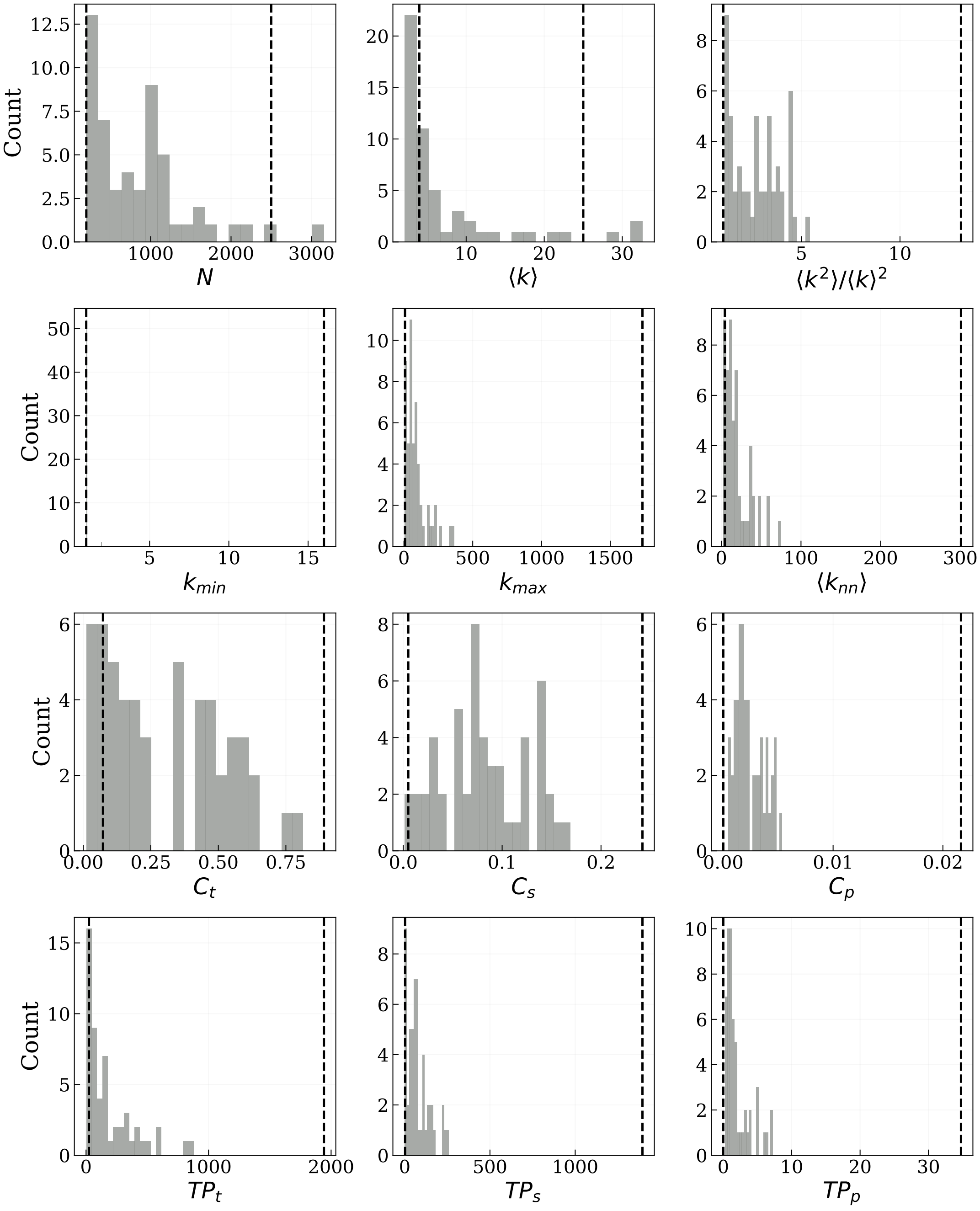

FIG. S8: Distributions of the topological properties of real networks. The dotted black lines in each panel indicate the minimum and maximum values of a given feature in the training dataset.

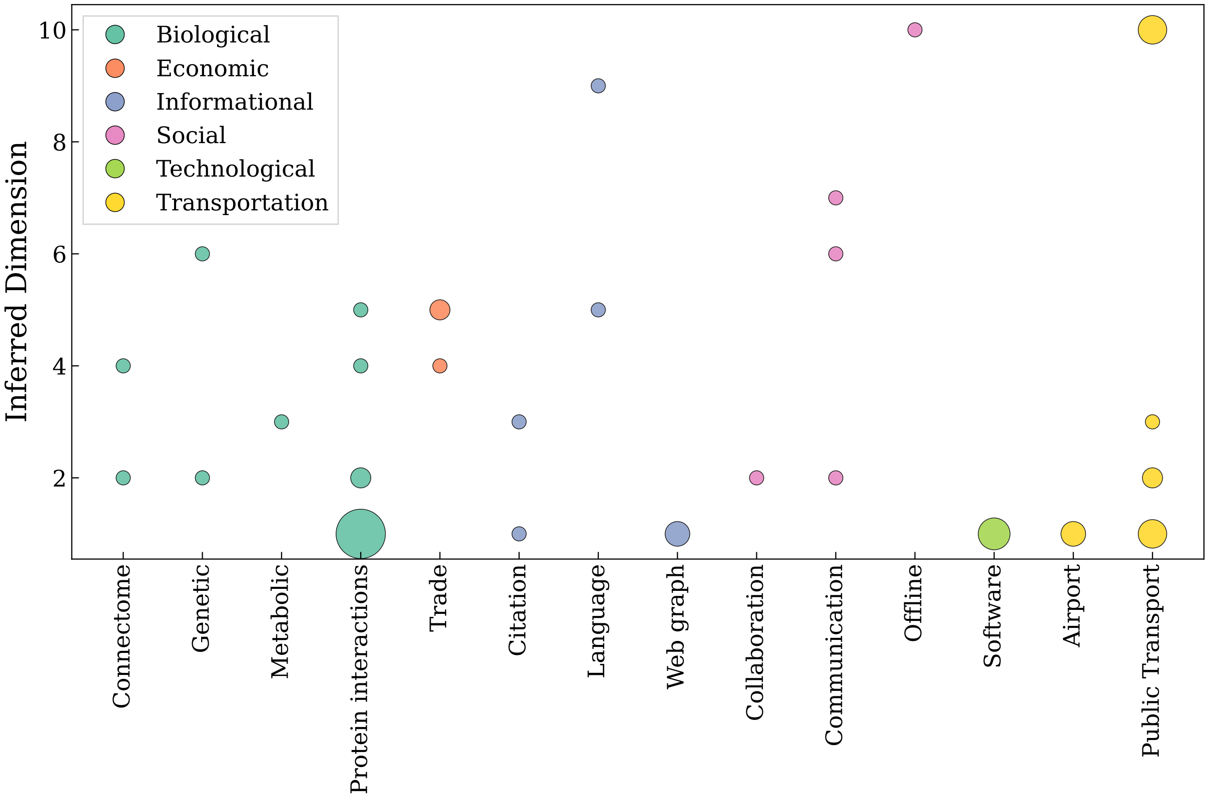

FIG. S9: Inferred dimension of real networks grouped by subdomain category.

TABLE S4: Properties of real networks, with network clustering, path and accuracy measures, and their dimensionality inferred with the DimNN model. Part 1 of 2.

Network	Domain	Subdomain	N	$\langle k\rangle$	$\langle k\rangle^2/\langle k\rangle^2$	$k_{\min}$	$k_{\max}$
Binary interactomes (various species; 2012)	Biological	Protein interactions	974	4.72	3.52	1	55
Uetz screen yeast interactome (2000)	Biological	Protein interactions	263	2.14	1.86	1	17
Reguly yeast interactome (2006)	Biological	Protein interactions	1213	4.21	2.11	1	40
Ito core yeast interactome (2001)	Biological	Protein interactions	426	2.45	3.44	1	56
Yu yeast interactomes (2008)	Biological	Protein interactions	964	3.09	3.86	1	85
C. elegans interactomes (2009)	Biological	Protein interactions	269	21.58	1.55	1	79
Binary interactomes (musculus mouse; 2012)	Biological	Protein interactions	537	3.83	2.99	1	59
Binary interactomes (homo sapiens; 2012)	Biological	Protein interactions	3155	3.58	3.31	1	131
Binary interactomes (musculus mouse; 2012)	Biological	Protein interactions	705	3.53	2.92	1	59
C. elegans interactomes (2009)	Biological	Protein interactions	2214	3.20	4.50	1	99
Binary interactomes (musculus mouse; 2012)	Biological	Protein interactions	890	2.99	4.50	1	76
C. elegans interactomes (scaffold, 2009)	Biological	Protein interactions	345	2.32	2.67	1	28
Yu yeast interactomes (2008)	Biological	Protein interactions	1647	3.06	3.82	1	89
C. elegans interactomes (genetic, 2009)	Biological	Protein interactions	683	4.52	2.39	1	56
C. elegans interactomes (WI-2004, 2009)	Biological	Protein interactions	1084	2.96	4.42	1	74
C. elegans interactomes (WI-2007, 2009)	Biological	Protein interactions	1108	2.71	4.42	1	84
Human2-C	Biological	Connectome	496	32.41	1.17	1	80
Human-M	Biological	Metabolic	1436	6.57	4.77	1	224
Malaria var DBLa HVR networks	Biological	Genetic	291	22.34	1.48	1	61
Malaria var DBLa HVR networks	Biological	Genetic	298	18.01	1.37	1	48
Cargoships	Economic	Trade	821	10.58	3.12	1	173
Atlas of Economic Complexity export network	Economic	Trade	774	4.59	2.70	1	43
Atlas of Economic Complexity export network	Economic	Trade	866	5.85	2.81	1	48
Garfield's citation networks (2001)	Informational	Citation	1024	9.60	3.95	1	232
AMiner citation network (2009)	Informational	Citation	1350	6.01	2.08	1	96
Roget's Thesaurus (1879)	Informational	Language	994	7.32	1.44	1	28
Bible-CO	Informational	Language	1707	10.61	3.92	2	364
WebKB graphs (1998)	Informational	Web graph	269	3.60	4.36	1	74
WebKB graphs (1998)	Informational	Web graph	280	3.95	2.75	1	57
WebKB graphs (1998)	Informational	Web graph	343	4.04	5.43	1	129
Friends-OFF	Social	Offline	2539	8.24	1.27	1	27
Friends-ON	Social	Communication	2000	16.10	2.72	1	273
EUEmail	Social	Communication	986	32.58	2.29	1	345
Jazz-CA	Social	Collaboration	199	29.23	1.51	1	189
Javax, Java, Jung, AppEngine dependencies (2010)	Technological	Software	1031	8.55	3.29	1	208
Software function-callgraphs (AbiWord, 2002)	Technological	Software	1035	3.32	3.27	1	89
Software function-callgraphs (VTK, 2002)	Technological	Software	771	3.52	3.66	1	83
Javax, Java, Jung, AppEngine dependencies (2010)	Technological	Software	435	5.99	2.59	1	109
Guava library dependencies (2012)	Technological	Software	457	4.05	3.85	1	62
World subways (2009)	Transportation	Public Transport	266	2.32	1.17	1	7
European airline network	Transportation	Airport	417	14.16	3.10	1	112
FAA Preferred Routes (2010)	Transportation	Airport	1226	3.93	1.87	1	34
US airport network (top 500; 2002)	Transportation	Airport	500	11.92	4.51	1	145
London Transport Network	Transportation	Public Transport	369	2.33	1.19	1	7
India bus routes (2016)	Transportation	Public Transport	1103	3.90	1.68	1	54
India bus routes (2016)	Transportation	Public Transport	1554	4.63	1.84	1	52
India bus routes (2016)	Transportation	Public Transport	1087	5.43	3.41	1	183
World subways (2009)	Transportation	Public Transport	217	2.41	1.22	1	9
World subways (2009)	Transportation	Public Transport	392	2.23	1.11	1	6
World subways (2009)	Transportation	Public Transport	299	2.38	1.22	1	8
World subways (2009)	Transportation	Public Transport	209	2.30	1.16	1	7
India bus routes (2016)	Transportation	Public Transport	1009	3.19	1.49	1	15
World subways (2009)	Transportation	Public Transport	433	2.19	1.13	1	8

TABLE S5: Properties of real networks, with network clustering, path and accuracy measures, and their dimensionality inferred with the DimNN model. Part 2 of 2.

Network	C_t	C_s	C_p	TP_t	TP_s	TP_p	Acc.	D
Binary interactomes (various species; 2012)	0.1220	0.1228	0.0031	77.7921	49.5511	1.5393	1.000	1
Uetz screen yeast interactome (2000)	0.0262	0.0251	0.0020	1.7500	2.1659	0.2455	1.000	1
Reguly yeast interactome (2006)	0.6015	0.0761	0.0020	227.0699	107.9266	3.2453	0.970	2
Ito core yeast interactome (2001)	0.1345	0.0875	0.0034	11.6167	15.8933	0.7123	1.000	1
Yu yeast interactomes (2008)	0.1217	0.1189	0.0015	39.2622	55.9388	1.0447	1.000	1
C. elegans interactomes (2009)	0.4635	0.1516	0.0021	149.4319	63.0049	0.7239	0.551	2
Binary interactomes (musculus mouse; 2012)	0.1646	0.1508	0.0027	40.5982	50.2391	1.2342	1.000	1
Binary interactomes (homo sapiens; 2012)	0.1933	0.1161	0.0013	301.0725	258.7704	3.5773	0.956	1
Binary interactomes (musculus mouse; 2012)	0.1744	0.1392	0.0023	50.8065	55.3945	1.2488	0.993	1
C. elegans interactomes (2009)	0.0728	0.0519	0.0010	82.7588	70.5024	1.2652	1.000	1
Binary interactomes (musculus mouse; 2012)	0.3684	0.0608	0.0015	82.2267	35.0576	0.9259	0.757	4
C. elegans interactomes (scaffold, 2009)	0.0803	0.0360	0.0034	6.8333	3.9875	0.4651	1.000	1
Yu yeast interactomes (2008)	0.1236	0.0762	0.0013	76.3197	64.8148	1.7078	1.000	1
C. elegans interactomes (genetic, 2009)	0.4244	0.0840	0.0019	153.5247	67.5384	1.5513	0.661	5
C. elegans interactomes (WI-2004, 2009)	0.0843	0.0689	0.0011	38.5890	40.9450	0.6395	1.000	1
C. elegans interactomes (WI-2007, 2009)	0.0727	0.0666	0.0015	30.0982	31.0904	0.7986	1.000	1
Human2-C	0.5378	0.1061	0.0009	323.3771	114.7546	0.7963	0.825	2
Human-M	0.5866	0.0770	0.0011	449.3374	177.3222	3.8313	0.987	3
Malaria var DBLa HVR networks	0.6054	0.1240	0.0018	179.7420	58.5849	1.0851	0.986	2
Malaria var DBLa HVR networks	0.4510	0.0710	0.0019	161.5944	31.7981	0.6912	0.870	6
Cargoships	0.5482	0.0873	0.0014	326.5759	131.8263	2.1144	0.775	5
Atlas of Economic Complexity export network	0.4882	0.0525	0.0022	109.7275	31.6048	1.5757	0.669	5
Atlas of Economic Complexity export network	0.5089	0.0759	0.0019	165.7158	57.9047	1.7043	0.734	4
Garfield's citation networks (2001)	0.3539	0.0842	0.0013	412.2120	162.5747	1.5339	1.000	1
AMiner citation network (2009)	0.3401	0.0841	0.0027	409.2855	152.9663	5.1329	0.789	3
Roget's Thesaurus (1879)	0.1964	0.0385	0.0018	307.8835	71.9862	2.8535	0.688	9
Bible-CO	0.6259	0.0347	0.0005	596.0701	122.7898	1.5247	0.828	5
WebKB graphs (1998)	0.3710	0.0731	0.0046	45.2202	20.3393	1.4145	1.000	1
WebKB graphs (1998)	0.4226	0.1367	0.0034	39.4806	28.7375	1.2093	0.689	1
WebKB graphs (1998)	0.2513	0.1365	0.0040	57.6453	44.9548	1.7253	1.000	1
Friends-OFF	0.1785	0.0282	0.0013	794.9442	144.4772	5.0352	0.712	10
Friends-ON	0.5241	0.0543	0.0007	878.8888	227.5659	1.8765	0.996	7
EUEmail	0.4735	0.1363	0.0009	572.1027	228.7467	1.1775	1.000	2
Jazz-CA	0.7568	0.1201	0.0010	133.8771	69.7319	0.6359	0.994	2
Javax, Java, Jung, AppEngine dependencies (2010)	0.4270	0.1397	0.0021	498.0842	235.9069	4.8104	1.000	1
Software function-callgraphs (AbiWord, 2002)	0.1398	0.0966	0.0037	78.2229	65.4463	2.6173	1.000	1
Software function-callgraphs (VTK, 2002)	0.1555	0.0939	0.0034	67.1126	47.4782	1.7499	1.000	1
Javax, Java, Jung, AppEngine dependencies (2010)	0.4683	0.1547	0.0041	154.3303	103.4080	3.3866	0.996	1
Guava library dependencies (2012)	0.6199	0.1396	0.0016	73.3721	55.9591	0.6418	0.976	1
World subways (2009)	0.0642	0.0284	0.0046	7.9000	4.9694	0.5619	0.389	10
European airline network	0.5508	0.1690	0.0018	165.0670	108.7857	1.2235	1.000	1
FAA Preferred Routes (2010)	0.1089	0.0527	0.0047	130.2926	70.0767	6.0710	0.930	1
US airport network (top 500; 2002)	0.8137	0.0907	0.0004	117.4738	106.0472	0.7758	1.000	1
London Transport Network	0.0528	0.0315	0.0023	9.9000	7.4528	0.5900	1.000	1
India bus routes (2016)	0.2469	0.0568	0.0053	225.6782	83.1205	7.2559	0.524	2
India bus routes (2016)	0.2359	0.0735	0.0030	359.0307	158.5868	6.9280	0.581	2
India bus routes (2016)	0.3333	0.0691	0.0032	329.8021	135.2038	6.3026	0.758	3
World subways (2009)	0.0453	0.0150	0.0047	6.0000	2.3667	0.5911	0.575	10
World subways (2009)	0.0111	0.0031	0.0040	2.6667	1.1250	1.0887	0.964	10
World subways (2009)	0.0393	0.0265	0.0040	6.8333	5.0270	0.9486	0.448	10
World subways (2009)	0.0128	0.0179	0.0015	1.6667	2.3889	0.2475	1.000	1
India bus routes (2016)	0.1282	0.1001	0.0044	95.3556	77.7124	3.9028	0.845	1
World subways (2009)	0.0303	0.0015	0.0022	6.6667	0.2571	0.5071	1.000	1

4. NETWORK GENERATION ALGORITHMS

Algorithm 1 $\mathbb{S}^D$ network generation algorithm

Input: Dimension D, number of nodes N, power-law exponent γ, average degree $\langle\kappa\rangle$, inverse temperature β
Output: Graph $G = (V, E)$ with nodes expressed in angular coordinates
$V \leftarrow \{\}$
$E \leftarrow \{\}$
$\kappa_0 \leftarrow \langle k\rangle(\gamma - 2)(1 - N^{-1})/[(\gamma - 1)(1 - N^{(2-\gamma)/(\gamma-1)})]$
$\kappa_c \leftarrow \kappa_0\, N^{1/(\gamma-1)}$
for $i = 1$ to N **do**
  $\kappa_i \sim \rho(\kappa) = \kappa^{-\gamma}\kappa_0^{\gamma-1}(\gamma - 1)/(1 - (\kappa_c/\kappa_0)^{1-\gamma})$
  $\theta_i \sim \text{Uniform}([0, 2\pi))$
  $V \leftarrow V \cup \{\theta_i\}$
end for
$\mu \leftarrow \beta\,\Gamma(D/2)\sin(D\pi/\beta)/(2\pi^{1+D/2}\langle k\rangle)$
for $i = 1$ to N **do**
  for $j = i + 1$ to N **do**
    $p_{ij} \leftarrow 1/(1 + [R\Delta\theta_{ij}/(\mu\kappa_i\kappa_j)^{1/D}]^{\beta})$
    $E \leftarrow E \cup \{(i, j)\}$ with probability p_{ij}
  end for
end for

Algorithm 2 Geometric randomization rewiring (D-GR)

Input: Graph $G = (V, E)$, dimension D, inverse temperature β
Output: Graph $G = (V, E)$ with rewired edges
for $t = 1$ to $10 \cdot |E|$ **do**
  $(i, j), (l, m) \sim \text{Uniform}(E)$ without replacement
  $\mathcal{L}_n/\mathcal{L}_c \leftarrow (\Delta\theta_{ij}\,\Delta\theta_{lm}/[\Delta\theta_{il}\,\Delta\theta_{jm}])^{\beta}$
  if $\mathcal{L}_n/\mathcal{L}_c > 1$ **then**
    $E \leftarrow E \cup \{(i, m), (l, j)\} \setminus \{(i, j), (l, m)\}$
  else
    $E \leftarrow E \cup \{(i, m), (l, j)\} \setminus \{(i, j), (l, m)\}$ with probability $\mathcal{L}_n/\mathcal{L}_c$
  end if
  if $|\mathcal{L}_n/\mathcal{L}_c - 1| < 10^{-6}$ **then**
    break
  end if
end for

5. VALIDATION WITH *D*-MERCATOR

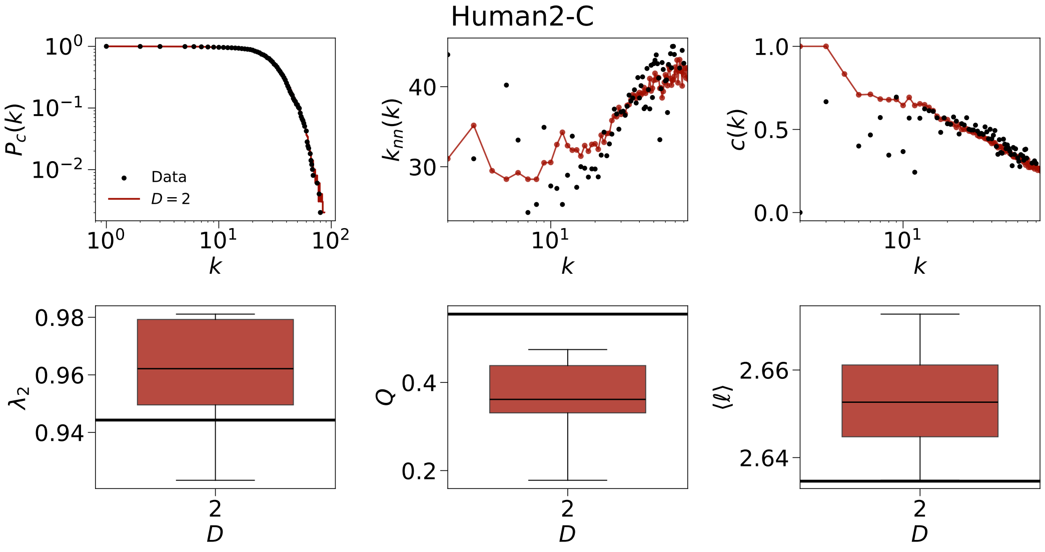

FIG. S10: **Validation for the Human2-C network.** We embed the Human2-C network with D-Mercator in dimension $D = 2$. We used the inferred positions to generate 10 synthetic networks and computed a set of topological descriptors: degree distribution $P(k)$; clustering spectrum $c(k)$; average nearest-neighbour degree $k_{\rm nn}(k)$; algebraic connectivity λ_2; modularity Q; and average shortest path length $\langle \ell \rangle$. Black markers show the target network compared to network surrogates. When applicable, error bars indicate variability across the 10 realizations.

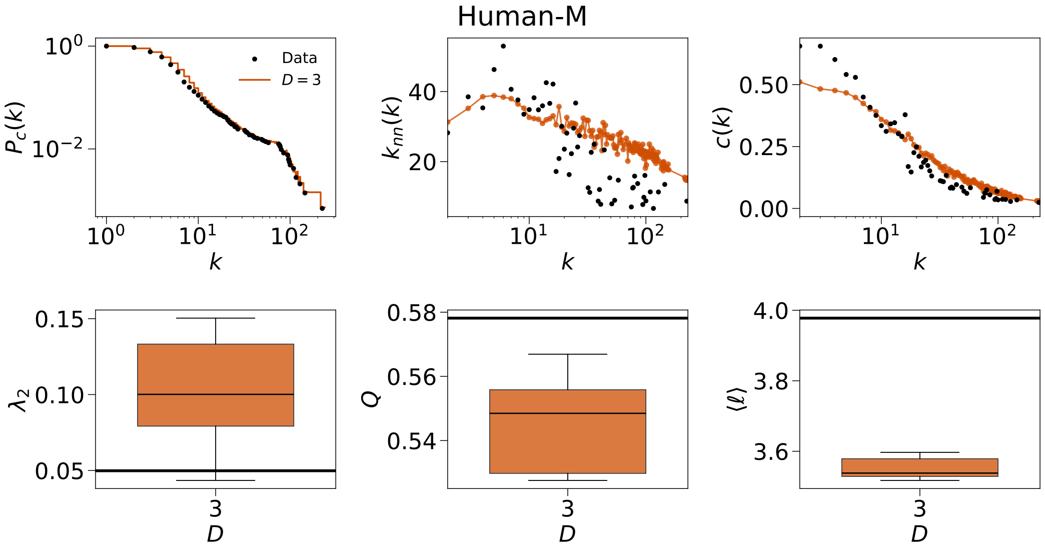

FIG. S11: **Validation for the Metabolic network.** We embed the Metabolic network with D-Mercator in dimension $D = 3$. We used the inferred positions to generate 10 synthetic networks and computed a set of topological descriptors: degree distribution $P(k)$; clustering spectrum $c(k)$; average nearest-neighbour degree $k_{\rm nn}(k)$; algebraic connectivity λ_2; modularity Q; and average shortest path length $\langle \ell \rangle$. Black markers show the target network compared to network surrogates. When applicable, error bars indicate variability across the 10 realizations.

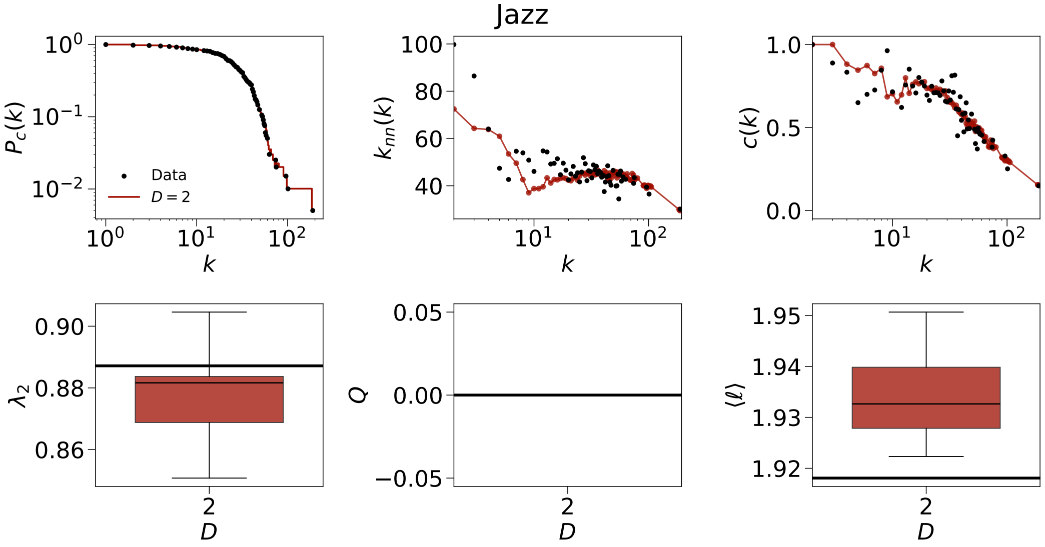

FIG. S12: **Validation for the Jazz network.** We embed the Jazz network with D-Mercator in dimension $D = 2$. We used the inferred positions to generate 10 synthetic networks and computed a set of topological descriptors: degree distribution $P(k)$; clustering spectrum $c(k)$; average nearest-neighbour degree $k_{\mathrm{nn}}(k)$; algebraic connectivity λ_2; modularity Q; and average shortest path length $\langle \ell \rangle$. Black markers show the target network compared to network surrogates. When applicable, error bars indicate variability across the 10 realizations.

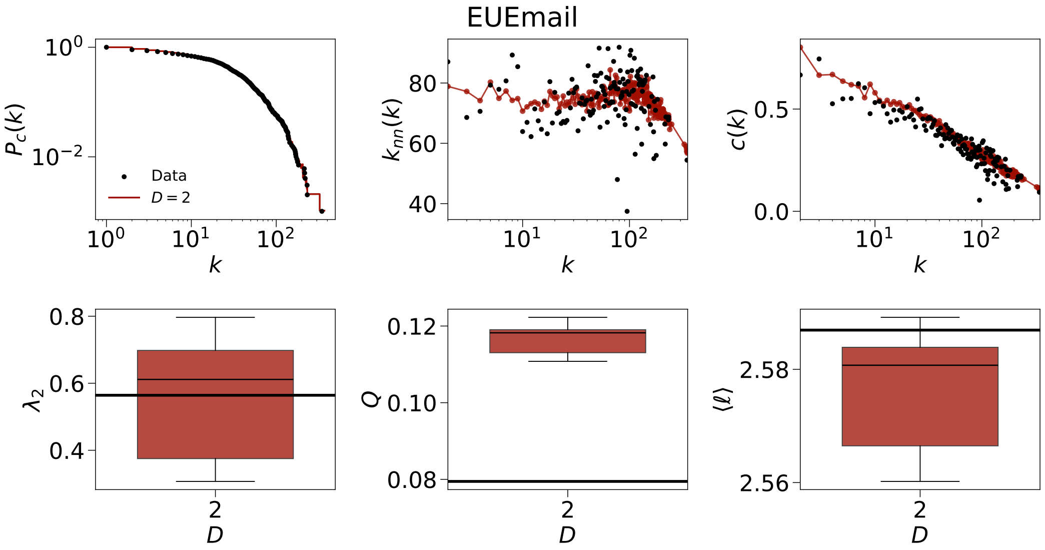

FIG. S13: **Validation for the EUEmail network.** We embed the EUEmail network with D-Mercator in dimension $D = 2$. We used the inferred positions to generate 10 synthetic networks and computed a set of topological descriptors: degree distribution $P(k)$; clustering spectrum $c(k)$; average nearest-neighbour degree $k_{\mathrm{nn}}(k)$; algebraic connectivity λ_2; modularity Q; and average shortest path length $\langle \ell \rangle$. Black markers show the target network compared to network surrogates. When applicable, error bars indicate variability across the 10 realizations.

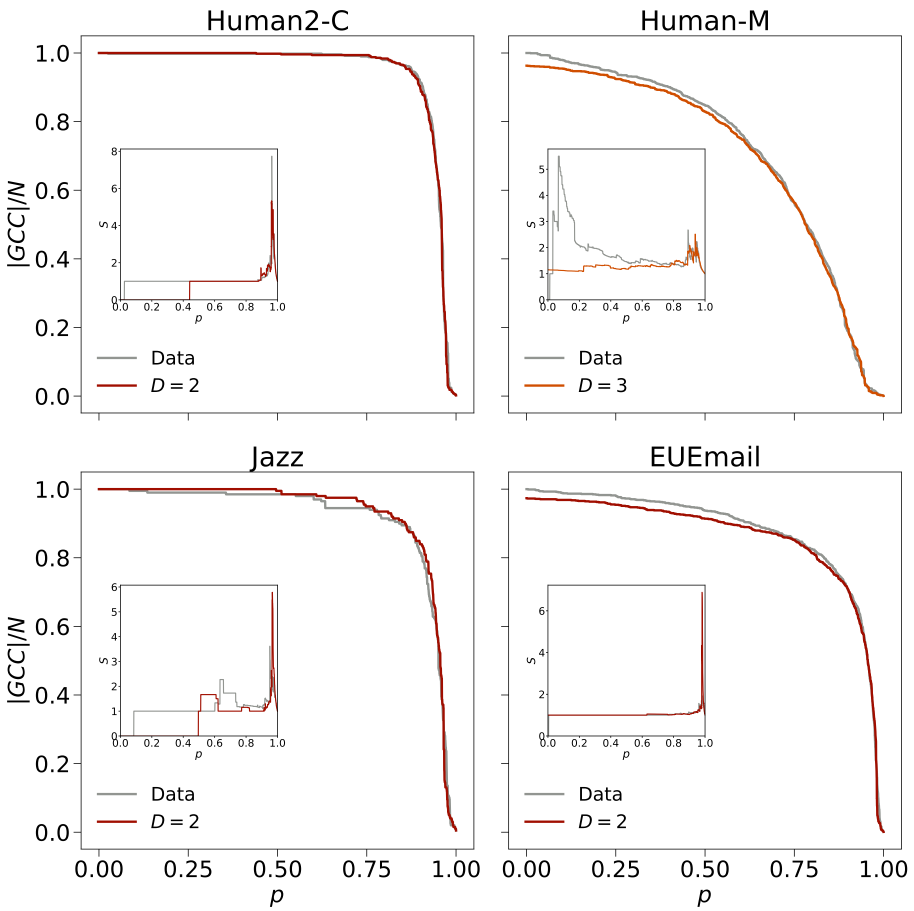

FIG. S14: **Validation of the percolation curves.** The relative size of the giant connected component ($|GCC|/N$) as a function of removed edges p. We compare the percolation curves between the original network and the synthetic network generated from the embedding obtained by D-Mercator, with the dimension predicted by DIMNN. In the inset of each panel, we plot the average cluster size S as the fraction of removed edges p.

SUPPLEMENTARY REFERENCES

[1] P. Almagro, M. Boguñá, and M. Á. Serrano, Nature Communications **13**, 6096 (2022)
[2] A. Ghasemian, H. Hosseinmardi, and A. Clauset, IEEE Transactions on Knowledge and Data Engineering **32**, 1722 (2019)
