# Supplementary material for: Chordless cycle filtrations for dimensionality detection in complex networks via topological data analysis
Source: Nat Commun. 2026 May 6;17:6105. doi: 10.1038/s41467-026-72687-z (PMC13358065; doi:10.1038/s41467-026-72687-z)
Supplement: Supplementary file 2 — Transparent Peer Review file [file 41467_2026_72687_MOESM2_ESM.pdf]

nature portfolio

Peer Review File

Chordless cycle filtrations for dimensionality detection in complex networks via topological data analysis

Corresponding Author: Professor M. Ángeles Serrano

This file contains all reviewer reports in order by version, followed by all author rebuttals in order by version.

Version 0:

Reviewer comments:

Reviewer #1

(Remarks to the Author)
This manuscript presents a novel topological data analysis (TDA) framework for estimating the intrinsic dimensionality of complex networks. The authors leverage chordless cycle densities, specifically triangles, squares, and pentagons, as edge weights to construct filtrations, from which extended persistence descriptors are computed. These topological features are then utilized in two complementary approaches: a k-nearest neighbors classifier applied to synthetic surrogate networks, and a pre-trained neural network (DimNN) that predicts dimensionality directly without retraining. The method is validated on a large collection of synthetic and real-world networks, demonstrating robust performance and providing a scalable, data-driven alternative to existing model-based dimensionality detection techniques. Despite promising results, several issues need addressing:

1 The paper lacks a clear explanation of the real-world relevance of the fundamental scientific problem of estimating network dimensionality.

2 The paper does not clearly define the scope or limitations of the proposed method. For instance, for networks that violate the geometric assumptions of the S^D/H^(D+1) model, such as classical ER random or scale-free networks, it remains unclear what results the method would yield and whether its predictions would still be meaningful.

3 Why is a long “lifetime” (i.e., high total persistence) of a cycle indicative of high-dimensional geometry? The authors should include a conceptual illustration, such as a comparison of persistence diagrams or total persistence between networks generated from high- versus low-dimensional models, to provide an intuitive understanding of this relationship.

4 The comparison with baseline methods should be strengthened. The current analysis focuses mainly on performance outcomes but lacks a quantitative evaluation of the relative efficiency of the two approaches.

5 The authors should perform a statistical significance test for the reported 0.68% performance improvement. While the accuracy increases from 82.32% to 83%, it is unclear whether this gain is statistically meaningful.

6 The authors should view dimensionality detection not as an endpoint but as a means to uncover deeper network properties. It is recommended to evaluate the proposed approach on one or two classic downstream tasks. For example, in low-dimensional spaces, link prediction may be easier because node similarities are more concentrated. The authors could examine whether the proposed TDA-based features achieve higher maximum capability (see PNAS Nexus, 3(3): pgae113, 2024) in link prediction and whether they capture higher-order structural information that traditional features overlook.

7 “Dimension D” should not be treated merely as an abstract numerical output of the model. The authors should systematically examine the relationship between the predicted dimension D and various classical network statistics. For example, does a higher D generally correspond to a lower global clustering coefficient, as theory suggests, given that the likelihood of forming closed triangular connections decreases in higher-dimensional spaces?

8 The current method constitutes a correlational analysis, predicting dimension from network structure. To strengthen the argument for causality, the authors are encouraged to design a more robust experiment. Specifically, they could select a real-world network, use DimNN to estimate its dimension D, and then reconstruct a new network using only the predicted D

and the original network's degree sequence based on the S^D model. A systematic comparison between the original and reconstructed networks should then be conducted across multiple global properties, such as clustering coefficient, community structure, and percolation threshold. If the reconstructed network successfully reproduces key characteristics of the original, it would provide strong evidence that the predicted dimension D is indeed an intrinsic and explanatory property of the network.

(Remarks on code availability)

Reviewer #2

(Remarks to the Author)
The paper presents a novel approach to dimensionality detection in complex networks, introducing a novel method based on Topological Data Analysis (TDA) that complements existing approaches. The authors propose a chordless cycle filtration scheme as a new way to extract and analyse topological features, leveraging extended persistence of cycles (triangles, squares and pentagons) as a key descriptor to characterise network structure. This method aims to capture the underlying geometric and topological properties of both synthetic and real-world networks, enabling more accurate dimensionality detection. Furthermore, the study integrates machine learning by training a multilayer perceptron on nearly 800,000 synthetic networks to predict network dimensionality. The trained model demonstrates strong generalisation capability, successfully transferring to real-world datasets. Importantly, experiments confirm that TDA-based features substantially enhance predictive performance when combined with conventional graph-theoretical descriptors.

Understanding the intrinsic dimensionality of complex networks is a fundamental challenge in applied network science, as it provides insights into the latent geometric structure that governs connectivity, flow, and dynamics within these systems. Accurate dimensionality detection allows researchers to better interpret how real-world networks organise and evolve, often revealing hidden constraints or symmetries that are not apparent from traditional graph-theoretic measures.

In this context, the present paper is highly relevant and timely: it addresses the dimensionality detection problem offering both conceptual and computational advances. Furthermore, this article represents a clear and successful attempt to bridge the gap between the fields of network science and TDA with the field of Machine Learning. In my opinion, attempts of this kind are key to ensure communication and exchange between scientific domains that too often find themselves tackling very similar scientific challenges, without seeking a truly interdisciplinary approach. This work does exactly that.

The manuscript is clearly written, methodologically sound, and demonstrates a deep understanding of both the theoretical underpinnings of TDA and its practical implications for data-driven network analysis.

I congratulate with the authors for their work. I only have a few minor comments that, if addressed, I believe would strengthen the results of this work along with their presentation.

First, although training on nearly 800 000 synthetic networks provides statistical power, the method's generalisation remains tied to the surrogate model family used for data generation. As seen in the mentioned reference (Almagro et al., Nat. Commun. 13, 6061 (2022)) study, matching degree distributions and sampling across parameter grids mitigates—but does not remove—model bias: real networks can deviate from the geometric soft configuration model's assumptions (in clustering, community structure, or long-range correlations).
A more detailed (Supplementary) analysis of how the training distribution spans the structural variability of real networks, or tests on out-of-distribution models, would therefore enhance confidence in the estimator's robustness.

Second, while the empirical performance of the extended-persistence features is compelling, the manuscript would benefit from stronger theoretical or mechanistic insight linking the total persistence of chordless cycles to latent dimensionality.

The 2022 work offered partial analytical intuition by deriving how small-cycle densities vary with dimension; a similar, even approximate, rationale for the persistence-based descriptors would substantially improve interpretability and clarify the limits of applicability.

A couple of minor comments regarding the presentation of the results:

I think, given the broad readership of the journal, moving up Figure 6 (now in the methods section), so that it is in between Figures 1 and 2, or potentially joined with Figure 2, could help the readers the definition of the chordless cycles-based filtration. I understand the narrative choices of the authors, I just think that Figure 6 is just as vital to quickly grasp the foundation of this work as Figures 1 and 2.

In the caption of Table 1, mention that DimNN is the neural network about to be presented in section 2.2.

Statistics of the training ensemble might be included in the main text or as supplementary information and mentioned in the main text.

In conclusion, I congratulate once more with the authors and I believe that this work is to be accepted for publication in the

journal upon completion of the improvements suggested.

(Remarks on code availability)
The code is well documented. Tutorials are easy to follow. The readme file is clear and very helpful.

Version 1:

Reviewer comments:

Reviewer #1

(Remarks to the Author)
I'd like to thank the authors for a thorough revision of their manuscript. They have done a commendable job addressing the previous feedback.

(Remarks on code availability)

Reviewer #2

(Remarks to the Author)
I congratulate with the authors for their work. They have successfully addressed all my comments and the improvements to the manuscript now make it a tangible contribution to the field. I recommend this article for publication.

(Remarks on code availability)
The code provides a clear README file ensuring proper running of the code.

Open Access This Peer Review File is licensed under a Creative Commons Attribution 4.0 International License, which permits use, sharing, adaptation, distribution and reproduction in any medium or format, as long as you give appropriate credit to the original author(s) and the source, provide a link to the Creative Commons license, and indicate if changes were made.
In cases where reviewers are anonymous, credit should be given to 'Anonymous Referee' and the source.
The images or other third party material in this Peer Review File are included in the article's Creative Commons license, unless indicated otherwise in a credit line to the material. If material is not included in the article's Creative Commons

license and your intended use is not permitted by statutory regulation or exceeds the permitted use, you will need to obtain permission directly from the copyright holder.
To view a copy of this license, visit https://creativecommons.org/licenses/by/4.0/

Resubmission of manuscript NCOMMS-25-74043

Chordless cycle filtrations for dimensionality detection in complex networks via topological data analysis

Aina Ferrà Marcús, Robert Jankowski, Meritxell Vila-Miñana, Carles Casacuberta, M. Ángeles Serrano

Reviewer #1

Comment 1.0: This manuscript presents a novel topological data analysis (TDA) framework for estimating the intrinsic dimensionality of complex networks. The authors leverage chordless cycle densities, specifically triangles, squares, and pentagons, as edge weights to construct filtrations, from which extended persistence descriptors are computed. These topological features are then utilized in two complementary approaches: a k-nearest neighbors classifier applied to synthetic surrogate networks, and a pre-trained neural network (DimNN) that predicts dimensionality directly without retraining. The method is validated on a large collection of synthetic and real-world networks, demonstrating robust performance and providing a scalable, data-driven alternative to existing model-based dimensionality detection techniques. Despite promising results, several issues need addressing:

Reply: We thank the Reviewer for their constructive feedback and pertinent suggestions, which have helped us improve substantially the content of our manuscript. We hope that the revisions and additional analyses detailed below satisfactorily address all of their concerns.

Comment 1.1: The paper lacks a clear explanation of the real-world relevance of the fundamental scientific problem of estimating network dimensionality.

Reply: We thank the Reviewer for this comment, and we agree. The effective dimensionality of complex phenomena has direct implications for structural properties, dynamical regimes, and the mechanisms that dominate the robustness, controllability, and criticality of real complex systems. In general, the effective dimensionality of the space in which a complex phenomenon unfolds is a recurring theme across the sciences. In statistical physics, spatial dimension strongly constrains scaling laws and often determines the universality class of critical, extended systems where many length scales matter. Even in the Ising model —the simplest widely used model of collective behavior and phase transitions, in which binary spins tend to align with their neighbors— dimensionality has a strong impact on critical behavior [1, 2]. For instance, in dimension $D = 1$, there is no finite temperature phase transition, whereas in $D = 2$ and higher, the Ising model undergoes a transition between an ordered and a disordered phase [1, 2]. Diffusion processes are also often studied through random walks on D-dimensional lattices, where the dimension controls return probabilities, exploration rates, and typical search/first-passage times [3-5]. In other areas of physics, such as string-theory settings, extra dimensions beyond the observed three are needed, but remain inaccessible because they are tightly compactified or because observable dynamics are confined to a lower-dimensional subset [6]. A similar idea appears in computer science: although data and interactions may live in a formally high-dimensional space, relevant configurations typically occupy a much smaller effective subspace, with important implications for both structure and dynamics [7, 8]. Dimensionality is also crucial in biology: transport and diffusion of molecules, encounter rates, and search processes depend sensitively on effective dimension [9, 10], and the structure of regulatory, neuronal, and ecological networks can enforce low-dimensional or genuinely multiscale dynamics, with different dimensions governing local versus global behavior [11]. However, dimensionality effects in networks remain underexplored, and our research aims to lay the groundwork for progress in this direction.

Action taken: We have revisited the Introduction section of the manuscript and added text to highlight the real-world relevance of the fundamental scientific problem of estimating network dimensionality. We have also added text to cite other non-equivalent approaches developed to measure network dimensionality within network science.

[1] Ising, E. (1925). Beitrag zur Theorie des Ferromagnetismus. Zeitschrift für Physik, 31, 253–258.
[2] Onsager, L. (1944). Crystal statistics. I. A two-dimensional model with an order-disorder transition. Physical Review, 65(3–4), 117–149.
[3] Redner, S. (2001). A guide to first-passage processes. Cambridge University Press.
[4] Dvoretzky, A. & Erdős, P. (1951). Some problems on random walk in space. In Proc. Second Berkeley Symp. Math. Statist. Probab. (pp. 353–367).
[5] Condamin, S., Bénichou, O., Tejedor, V., Voituriez, R. & Klafter, J. (2007). First-passage times in complex scale-invariant media. Nature, 450(7166), 77–80.
[6] Polchinski, J. (1998). String Theory. Cambridge University Press.
[7] Tenenbaum, J. B., de Silva, V. & Langford, J. C. (2000). A global geometric framework for nonlinear dimensionality reduction. Science, 290(5500), 2319–2323.
[8] Belkin, M. & Niyogi, P. (2003). Laplacian eigenmaps for dimensionality reduction and data representation. Neural Computation, 15(6), 1373–1396.
[9] Codling, E. A., Plank, M. J. & Benhamou, S. (2008). Random walk models in biology. Journal of the Royal Society Interface, 5(25), 813–834.
[10] von Hippel, P. H. & Berg, O. G. (1989). Facilitated target location in biological systems. Journal of Biological Chemistry, 264(2), 675–678.
[11] Peach, R. L., Arnaudon, A. & Barahona, M. (2022). Relative, local and global dimension in complex networks. Nature Communications, 13, 3088.

Comment 1.2: The paper does not clearly define the scope or limitations of the proposed method. For instance, for networks that violate the geometric assumptions of the S^D/H^{D+1} model, such as classical ER random or scale-free networks, it remains unclear what results the method would yield and whether its predictions would still be meaningful.

Reply: Indeed, our focus is on real networks and many real networks are well described by the $\mathbb{S}^D/\mathbb{H}^{D+1}$ model. It has been shown that this model can reproduce many topological properties observed in real systems, such as heterogeneous and power-law degree distributions —thus, it is appropriate for scale-free networks—, high clustering coefficient, different forms of self-similarity, the small-world effect, and more [1–3]. Thus, we believe that our assumption is well justified given the extensive empirical evidence supporting the model's ability to capture the structural organization of a wide range of complex networks.

We agree that there exist real networks that do not strictly follow the model, as indicated by the Reviewer. For instance, in this paper, we focus on the geometric region, i.e., when $\beta > D$. However, some networks might reside in the weakly geometric region $\beta < D$ [4, 5] (the Configuration Model is recovered in the limit $\beta \to 0$) where clustering is lower, for which our current approach does not account. We also think it is important to point out this limitation. However, we leave understanding the interplay between dimensionality and the weakly and non-geometric regions for future work.

Action taken: We added a paragraph in the Discussion section pointing out the limitations of the current approach, as well as future work in these directions.

[1] Serrano, M. Á., Krioukov, D. & Boguñá, M. (2008). Self-similarity of complex networks and hidden metric spaces. Physical Review Letters, 100(7), 078701.
[2] Papadopoulos, F., Kitsak, M., Serrano, M. Á., Boguñá, M. & Krioukov, D. (2012). Popularity

versus similarity in growing networks. Nature, 489(7417), 537–540.
[3] Boguñá, M., Bonamassa, I., De Domenico, M., Havlin, S., Krioukov, D. & Serrano, M. Á. (2021). Network Geometry. Nature Reviews Physics, 3(2), 114–135.
[4] van der Kolk, J., Serrano, M. Á. & Boguñá, M. (2022). An anomalous topological phase transition in spatial random graphs. Communications Physics, 5(1), 245.
[5] van der Kolk, J., Serrano, M. Á. & Boguñá, M. (2024). Random graphs and real networks with weak geometric coupling. Physical Review Research, 6(1), 013337.

Comment 1.3: Why is a long "lifetime" (i.e., high total persistence) of a cycle indicative of high-dimensional geometry? The authors should include a conceptual illustration, such as a comparison of persistence diagrams or total persistence between networks generated from high- versus low-dimensional models, to provide an intuitive understanding of this relationship.

Reply: We thank the Reviewer for this valuable remark, which has led to a clarification. We do not claim that high total persistence is indicative of high-dimensional geometry. Figures S1 and S2 in the Supplementary Information show that total persistence can take a broad range of values across different dimensions, and high-dimensional networks tend to display lower total persistence. Fig. R1 shows total persistence versus dimension for the different density filtrations using the SynNet database. We observe an inverse association between total persistence and embedding dimension, most pronounced in the case of square-density and pentagon-density filtrations.

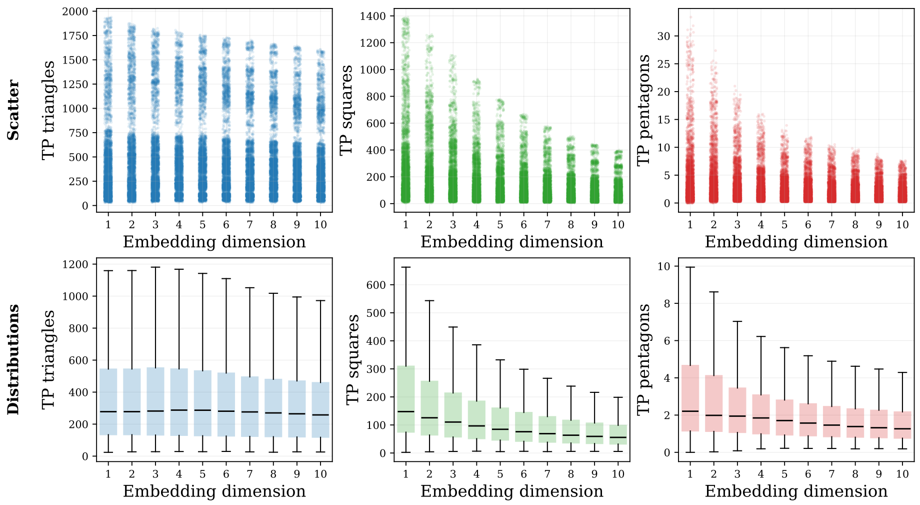

Figure R1: Inverse relationship between network dimension (x-axis) and total extended persistence of cycles (y-axis) for the three filtrations considered in the article: triangles (left), squares (middle), and pentagons (right). *Top row:* Jittered scatter plots; *Bottom row:* Boxplots summarizing the distribution within each dimension. These distributions have been obtained from a sample of 50 000 graphs from the SynNet database, uniformly distributed with respect to dimension.

Action taken: Fig. R1 has ben added to the Supplementary Information as Fig. S3 and has been referenced in page 6 of the main text of the article, including Pearson correlation coefficients.

Comment 1.4: The comparison with baseline methods should be strengthened. The current analysis focuses mainly on performance outcomes but lacks a quantitative evaluation of the relative efficiency of the two approaches.

Reply: Currently, we can list three different approaches for detecting dimensions within the geometric network models ($\mathbb{S}^D/\mathbb{H}^{D+1}$ model) approach: (i) Almagro et al. [1] used cycle statistics to infer a network's dimension; (ii) Jankowski et al. [2] proposed D-Mercator, an embedding algorithm to

map networks into multidimensional hyperbolic spaces, and based on greedy routing and community concentration infer dimension, and (iii) this work proposes to leverage TDA to detect dimension. In fact, in this work, we used TDA in two ways: first, from persistence descriptors of the generated network ensemble, and second, with a trained neural network DimNN. In Fig. 3 and Fig. 4 of the main paper we report a comparison of the inferred dimension and the true dimension over synthetic networks, with very good results, and in Table 1 we report the inferred dimension using the mean densities of chordless cycles, the TDA persistent homology from chordless cycle density filtrations, and DimNN on real networks, and the results are consistent.

With DimNN, we addressed the computationally expensive task of generating a network ensemble for every real network required by other methods. The initial high cost of neural network training allows us later to rapidly infer the effective dimension in subsequent studies without retraining. We are not aware of any other method in the literature that tackles this problem. However, by releasing the SynNet dataset, we invite other researchers to develop methods for detecting network dimensionality.

[1] Almagro, P., Boguñá, M. & Serrano, M. Á. (2022). Detecting the ultra low dimensionality of real networks. Nature Communications, 13(1), 6096.
[2] Jankowski, R., Allard, A., Boguñá, M. & Serrano, M. Á. (2023). The D-Mercator method for the multidimensional hyperbolic embedding of real networks. Nature Communications, 14(1), 7585.

Comment 1.5: The authors should perform a statistical significance test for the reported 0.68% performance improvement. While the accuracy increases from 82.32% to 83%, it is unclear whether this gain is statistically meaningful.

Reply: Following the Reviewer's suggestion, we performed paired t-tests comparing the validation accuracies obtained with the combined feature vector against those obtained with topological features only and density-based features only.

We observe that in scenarios (i)–(iii) the combined representation yields statistically significant improvements over both baselines. When the second moment information is included in the density-based feature vector (scenarios (iv)–(vi)), the improvement of the combined representation over the mean-density-only baseline is no longer statistically significant, whereas the improvement over the topology-only baseline remains statistically significant. These results are summarized in Fig. R2, where significance markers are reported.

Finally, we emphasize that the proposed pipeline is intended as a proof of concept: our primary goal is to demonstrate that topological descriptors can provide complementary predictive information. A more extensive hyperparameter search and architectural optimization could likely further improve absolute accuracies, but this is beyond the scope of the present work.

Action taken: The former Fig. 4 in the main text has been replaced by Fig. R2 (now Fig. 5 in the article), and an explanation of the statistical tests has been added.

Comment 1.6: The authors should view dimensionality detection not as an endpoint but as a means to uncover deeper network properties. It is recommended to evaluate the proposed approach on one or two classic downstream tasks. For example, in low-dimensional spaces, link prediction may be easier because node similarities are more concentrated. The authors could examine whether the proposed TDA-based features achieve higher maximum capability (see PNAS Nexus, 3(3): pgae113, 2024) in link prediction and whether they capture higher-order structural information that traditional features overlook.

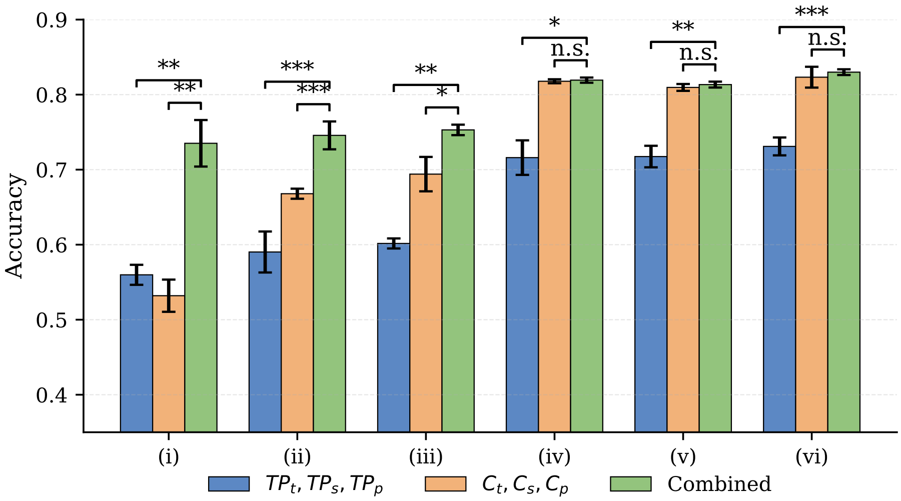

Figure R2: Validation accuracies after five repetitions of DimNN using the SynNet database. *Blue:* Accuracies obtained using total persistences TP_t, TP_s, TP_p, plus cumulative features. *Orange:* Accuracies using average cycle densities C_t, C_s, C_p, plus cumulative features *Green:* Accuracies obtained combining total persistences and mean cycle densities plus cumulative features. Horizontal brackets indicate paired comparisons between the combined model and each baseline; significance corresponds to paired t-tests with $p < 0.05$ (*), $p < 0.01$ (**), and $p < 0.001$ (***), while "n.s." indicates a non-significant difference.

Reply: We fully agree that dimensionality detection is a key step in modeling networks and data. Our paper focuses on this point by proposing a TDA and an end-to-end machine learning pipeline to facilitate it. It is true that the network dimension is not only an abstract numerical value, but it also impacts downstream tasks.

In our previous work [1], examining network communities, we observed a strong correlation between community separation and embedding dimension. For instance, in Fig. 4 of [1], the best dimension was found to be $D = 2$. At the same time, as shown in Supplementary Fig. S27 of [1], we observed good agreement in validating the topological properties of real networks across the optimal dimension and other tested dimensions. Thus, even though local properties might be well recovered with a larger dimension, the large-scale organization of the network may differ. Désy et al. also highlighted the importance of network dimension in modeling community structure in hyperbolic spaces [2]. Another task, named navigability, i.e., routing information using local information from embeddings, also corroborates a strong correlation with network dimension (Fig. 3a-d of [1]). Moreover, the impact of dimensionality on the downstream task was partially explored in [3]. We compared embeddings of unipartite and bipartite networks in $D = 1$ and $D = 2$ for node classification and link prediction tasks. In some real networks, performance is higher for $D = 2$, which may be related to the optimal dimension of these networks. However, in [3] we intentionally analyze the simplest cases ($D = 1$ and $D = 2$), to demonstrate that our method remains competitive with alternatives that use much higher dimensions.

Persistent homology has been shown to be effective for predicting missing links [4, 5], performing on par or sometimes better than network topology features. It would indeed be interesting to incorporate our chordless cycle filtration into the pipeline and potential future work in this direction. Kitsak et al. have shown that hyperbolic space is suitable for the link prediction task, especially when the fraction of missing links is large. They focused on the $\mathbb{S}^1/\mathbb{H}^2$ model, i.e., the simplest one-dimensional case. Their proposed embedder, HyperLink accounts for a fraction of missing links by using effective connection probability. However, it can only embed networks in $D = 1$. In our previous work, we have proposed

D-Mercator [1], an extension of Mercator [7] to higher-dimensional hyperbolic spaces. However, in its current form, it does not support calculating the effective connection probability for a given fraction of removed edges. We agree that this is an interesting research problem, and we are currently exploring the impact of dimensionality on the link prediction task in another project.

[1] Jankowski, R., Allard, A., Boguñá, M. & Serrano, M. Á. (2023). The D-Mercator method for the multidimensional hyperbolic embedding of real networks. Nature Communications, 14(1), 7585.
[2] Désy, B., Desrosiers, P. & Allard, A. (2023). Dimension matters when modeling network communities in hyperbolic spaces. PNAS Nexus, 2(5), pgad136.
[3] Jankowski, R., Aliakbarisani, R., Serrano M. Á. et al. (2026). Mapping bipartite networks into multidimensional hyperbolic spaces. Commun Phys 9, 35.
[4] Yan, Z., Ma, T., Gao, L., Tang, Z. & Chen, C. (2021). Link prediction with persistent homology: An interactive view. Proceedings of the 38th International Conference on Machine Learning, in Proceedings of Machine Learning Research 139:11659-11669.
[5] Junwon You, Eunwoo Heo, & Jae-Hun Jung (2026). PHLP: Sole persistent homology for link prediction – interpretable feature extraction. Neurocomputing, 665, 132147.
[6] Kitsak, M., Voitalov, I. & Krioukov, D. (2020). Link prediction with hyperbolic geometry. Physical Review Research, 2(4), 043113.
[7] García-Pérez, G., Allard, A., Serrano, M. Á. & Boguñá, M. (2019). Mercator: Uncovering faithful hyperbolic embeddings of complex networks. New Journal of Physics, 21(12), 123033.

Comment 1.7: "Dimension D" should not be treated merely as an abstract numerical output of the model. The authors should systematically examine the relationship between the predicted dimension D and various classical network statistics. For example, does a higher D generally correspond to a lower global clustering coefficient, as theory suggests, given that the likelihood of forming closed triangular connections decreases in higher-dimensional spaces?

Reply: We agree that dimension affects network topology. In our previous work [1], we quantified how clustering and other cycle statistics vary with the model dimension. In Fig. R3B, for $\beta \to \infty$, the (edge) triangle-based clustering measure C_t decreases with the model dimension. This follows from distance concentration in high dimensions: as D increases, pairwise angular distances concentrate around $\pi/2$, so neighbors become less likely to share common neighbors and triangle closure vanishes as $D \to \infty$. Figure R3C shows the corresponding behavior for chordless squares, which can exhibit a maximum at intermediate D depending on γ, and Fig. R3D reports the analogous trend for chordless pentagons.

As discussed in our reply to Comment 1.1, dimensionality has concrete implications across physical and biological systems and should therefore not be viewed as a purely abstract output. In networks, dimensionality has direct consequences for topology and function; for example, it impacts navigability [2] and the ability of geometric models to capture community structure [3]. Our framework provides an estimate of the intrinsic dimension that is most consistent with the observed network structure within the $\mathbb{S}^D/\mathbb{H}^{D+1}$ modeling paradigm.

[1] Almagro, P., Boguñá, M. & Serrano, M. Á. (2022). Detecting the ultra low dimensionality of real networks. Nature Communications, 13(1), 6096.
[2] Jankowski, R., Allard, A., Boguñá, M. & Serrano, M. Á. (2023). The D-Mercator method for the multidimensional hyperbolic embedding of real networks. Nature Communications, 14(1), 7585.
[3] Désy, B., Desrosiers, P. & Allard, A. (2023). Dimension matters when modeling network communities in hyperbolic spaces. PNAS Nexus, 2(5), pgad136.

Comment 1.8: The current method constitutes a correlational analysis, predicting dimension from network structure. To strengthen the argument for causality, the authors are encouraged to design a

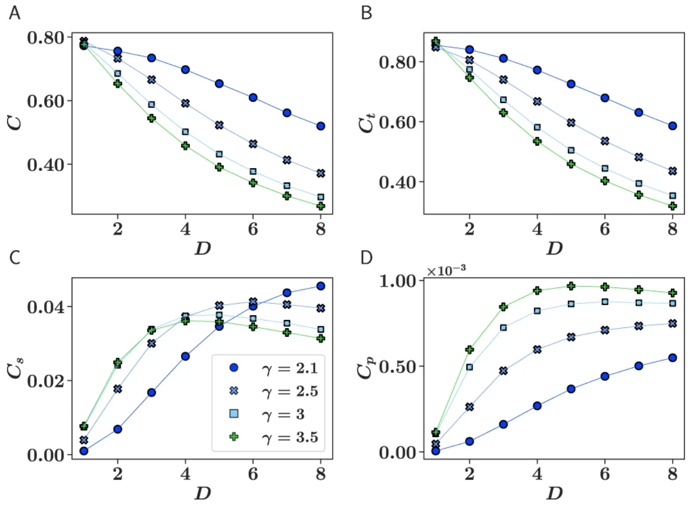

Figure R3: Maximum value of node clustering (A), and mean density of edge triangles (B), squares (C), and pentagons (D), as a function of the dimension D and power law exponent γ in synthetic networks generated by the $\mathbb{S}^D$ model with $\beta \to \infty$. Figure taken from [1].

more robust experiment. Specifically, they could select a real-world network, use DimNN to estimate its dimension D, and then reconstruct a new network using only the predicted D and the original network's degree sequence based on the S^D model. A systematic comparison between the original and reconstructed networks should then be conducted across multiple global properties, such as clustering coefficient, community structure, and percolation threshold. If the reconstructed network successfully reproduces key characteristics of the original, it would provide strong evidence that the predicted dimension D is indeed an intrinsic and explanatory property of the network.

Reply: We thank the Reviewer for this opportunity to improve the paper. We conducted experiments using D-Mercator. We selected real networks and embedded each of them in the dimension predicted by DimNN. Next, we used the inferred nodes' positions to generate a set of surrogate networks. For each synthetic network, we computed multiple topological properties and compared them with those of the selected real networks. Figures R4, R5, R6, and R7 show the degree distribution, clustering spectrum, average neighborhood degree for each degree class, algebraic connectivity, modularity, and average shortest path length. To find network communities, we ran the label propagation algorithm [2].

First, we can observe good agreement in the degree distribution, clustering spectrum, and average neighborhood degree spectrum for the embedding in the predicted dimension. Furthermore, the algebraic connectivity of the surrogate networks, in most cases, agrees with the ground truth, as does the average shortest path. Highlighting the dimension predicted by DimNN is therefore particularly informative. On the other hand, the modularity of the surrogate networks shows a less clear picture. It may be related to the algorithm used to detect topological communities. Here, we used a label propagation algorithm. However, other community detection methods might be more suitable.

Moreover, we studied bond percolation. In Fig. R8 we plot the relative size of the giant connected component as a function of removed edges. The grey curves correspond to the original networks and the colored ones to the synthetic networks generated from the embeddings obtained with D-Mercator in the dimension predicted by DimNN. One can observe good agreement for the percolation threshold (confirmed by the inset figures showing the average cluster size).

Action taken: We have added the validation of the DimNN inferred dimension using D-Mercator surrogates to the Supplementary Information (Figs. S10–S13). We have also added one paragraph at the end of the Results section to refer to the results of this experiment.

[1] Jankowski, R., Allard, A., Boguñá, M. & Serrano, M. Á. (2023). The D-Mercator method for the multidimensional hyperbolic embedding of real networks. Nature Communications, 14(1), 7585.
[2] Cordasco, G. & Gargano, L. (2010). Community detection via semi-synchronous label propagation algorithms. In 2010 IEEE Workshop on Business Applications of Social Network Analysis, pp. 1–8.

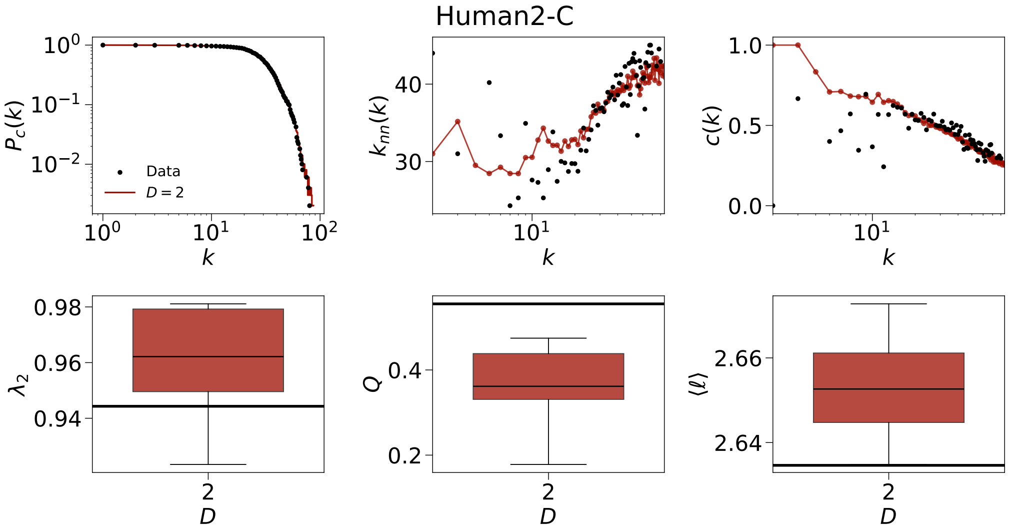

Figure R4: **Validation for the Human2-C network.** We embed the Human2-C network with D-Mercator in dimension $D = 2$. We used the inferred positions to generate 10 synthetic networks and computed a set of topological descriptors: degree distribution $P(k)$; clustering spectrum $c(k)$; average nearest-neighbour degree $k_{\rm nn}(k)$; algebraic connectivity λ_2; modularity Q; and average shortest path length $\langle \ell \rangle$. Black markers show the target network compared to network surrogates. When applicable, error bars indicate variability across the 10 realizations.

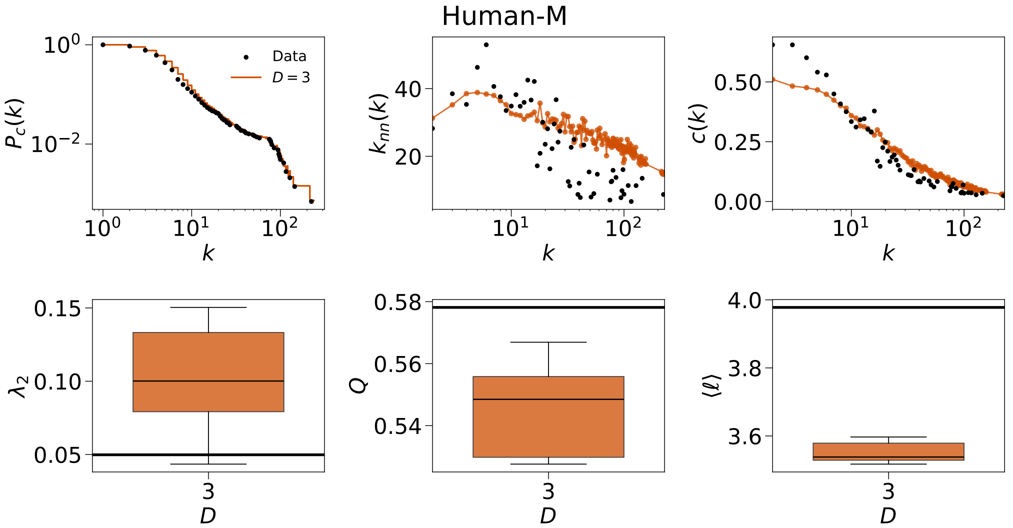

Figure R5: **Validation for the Metabolic network.** We embed the Metabolic network with D-Mercator in dimension $D = 3$. We used the inferred positions to generate 10 synthetic networks and computed a set of topological descriptors: degree distribution $P(k)$; clustering spectrum $c(k)$; average nearest-neighbour degree $k_{\rm nn}(k)$; algebraic connectivity λ_2; modularity Q; and average shortest path length $\langle \ell \rangle$. Black markers show the target network compared to network surrogates. When applicable, error bars indicate variability across the 10 realizations.

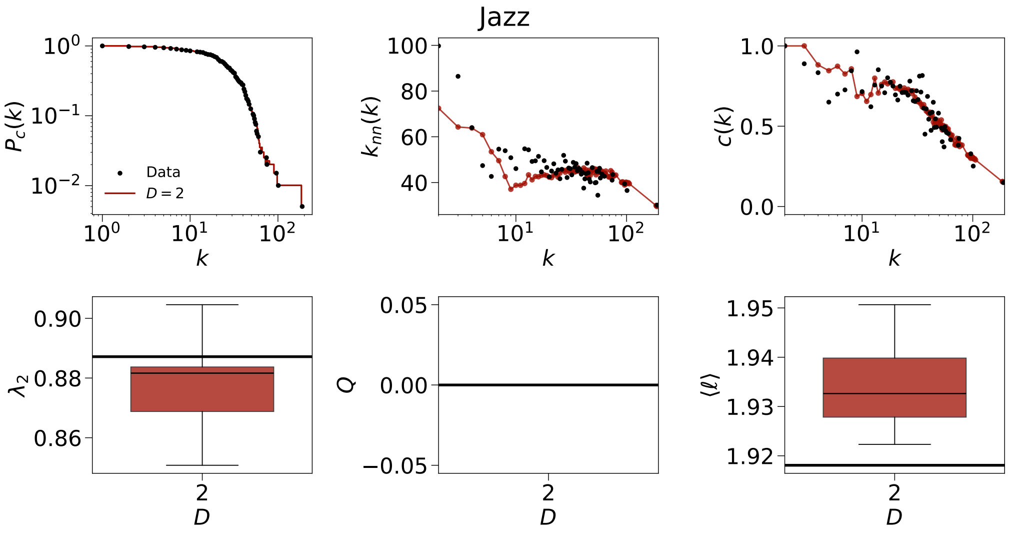

Figure R6: **Validation for the Jazz network.** We embed the Jazz network with D-Mercator in dimension $D = 2$. We used the inferred positions to generate 10 synthetic networks and computed a set of topological descriptors: degree distribution $P(k)$; clustering spectrum $c(k)$; average nearest-neighbour degree $k_{\mathrm{nn}}(k)$; algebraic connectivity λ_2; modularity Q; and average shortest path length $\langle \ell \rangle$. Black markers show the target network compared to network surrogates. When applicable, error bars indicate variability across the 10 realizations.

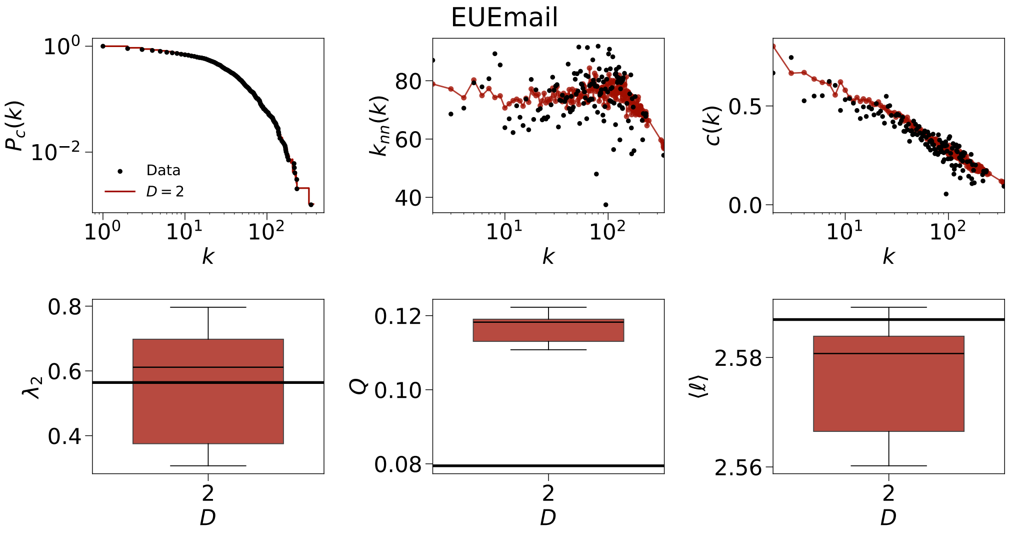

Figure R7: **Validation for the EUEmail network.** We embed the EUEmail network with D-Mercator in dimension $D = 2$. We used the inferred positions to generate 10 synthetic networks and computed a set of topological descriptors: degree distribution $P(k)$; clustering spectrum $c(k)$; average nearest-neighbour degree $k_{\mathrm{nn}}(k)$; algebraic connectivity λ_2; modularity Q; and average shortest path length $\langle \ell \rangle$. Black markers show the target network compared to network surrogates. When applicable, error bars indicate variability across the 10 realizations.

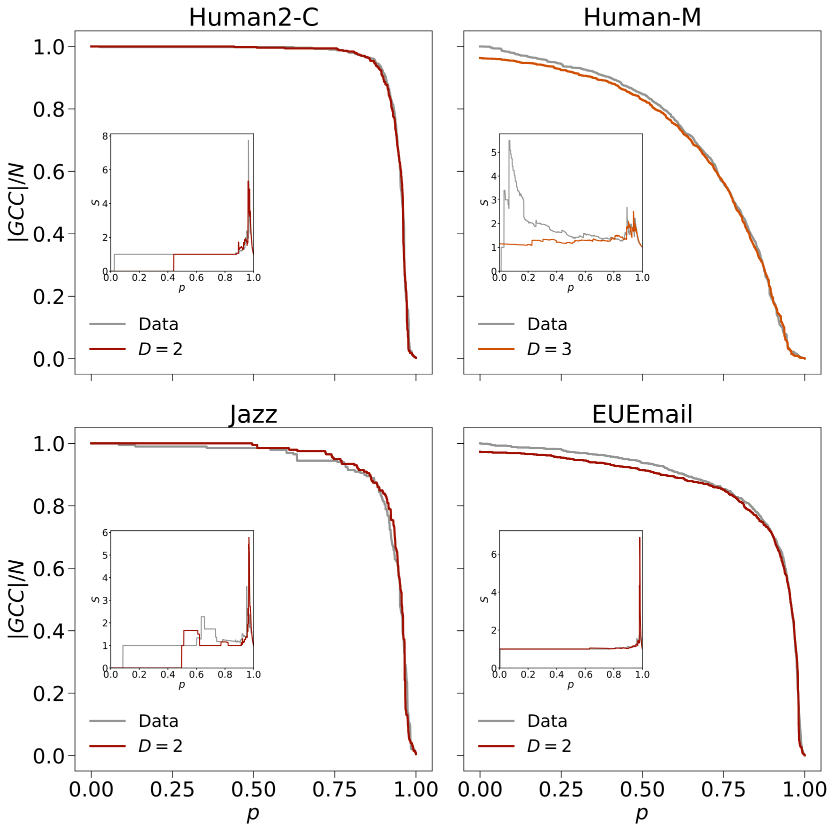

Figure R8: **Validation of the percolation curves.** The relative size of the giant connected component ($|\text{GCC}|/N$) as a function of removed edges p. We compare the percolation curves between the original network and the synthetic network generated from the embedding obtained by D-Mercator, with the dimension predicted by DimNN. In the inset of each panel we plot the average cluster size S as the fraction of removed edges p.

Reviewer #2

Comment 2.0: The paper presents a novel approach to dimensionality detection in complex networks, introducing a novel method based on Topological Data Analysis (TDA) that complements existing approaches. The authors propose a chordless cycle filtration scheme as a new way to extract and analyse topological features, leveraging extended persistence of cycles (triangles, squares and pentagons) as a key descriptor to characterise network structure. This method aims to capture the underlying geometric and topological properties of both synthetic and real-world networks, enabling more accurate dimensionality detection. Furthermore, the study integrates machine learning by training a multilayer perceptron on nearly 800,000 synthetic networks to predict network dimensionality. The trained model demonstrates strong generalisation capability, successfully transferring to real-world datasets. Importantly, experiments confirm that TDA-based features substantially enhance predictive performance when combined with conventional graph-theoretical descriptors.

Understanding the intrinsic dimensionality of complex networks is a fundamental challenge in applied network science, as it provides insights into the latent geometric structure that governs connectivity, flow, and dynamics within these systems. Accurate dimensionality detection allows researchers to better interpret how real-world networks organise and evolve, often revealing hidden constraints or symmetries that are not apparent from traditional graph-theoretic measures.

In this context, the present paper is highly relevant and timely: it addresses the dimensionality detection problem offering both conceptual and computational advances. Furthermore, this article represents a clear and successful attempt to bridge the gap between the fields of network science and TDA with the field of Machine Learning. In my opinion, attempts of this kind are key to ensure communication and exchange between scientific domains that too often find themselves tackling very similar scientific challenges, without seeking a truly interdisciplinary approach. This work does exactly that.

The manuscript is clearly written, methodologically sound, and demonstrates a deep understanding of both the theoretical underpinnings of TDA and its practical implications for data-driven network analysis.

I congratulate with the authors for their work. I only have a few minor comments that, if addressed, I believe would strengthen the results of this work along with their presentation.

Reply: We thank the Reviewer for their positive assessment of our work and for their constructive suggestions. We have carefully implemented all requested improvements and believe that the revised manuscript has significantly benefited from the Reviewer's comments.

Comment 2.1: First, although training on nearly $800\,000$ synthetic networks provides statistical power, the method's generalisation remains tied to the surrogate model family used for data generation. As seen in the mentioned reference (Almagro et al., Nat. Commun. 13, 6061 (2022)) study, matching degree distributions and sampling across parameter grids mitigates—but does not remove—model bias: real networks can deviate from the geometric soft configuration model's assumptions (in clustering, community structure, or long-range correlations). A more detailed (Supplementary) analysis of how the training distribution spans the structural variability of real networks, or tests on out-of-distribution models, would therefore enhance confidence in the estimator's robustness.

Reply: We agree that some properties of real networks may not be captured by our geometric network model. When generating a set of $800\,000$ synthetic networks, we made sure that they cover a wide range of network properties. As shown in Supplementary Figure S8, we plotted the distributions of topological properties of real networks, along with the minimum and maximum values from our

SynNet dataset. One can see that most real networks fall within these ranges, with a few exceptions. For instance, some real networks are larger than synthetic ones. A key advantage of our framework is that we can easily generate additional synthetic networks and expand the training ensemble in targeted regions of the descriptor space whenever new real networks fall outside the previously sampled range.

We conducted an experiment with D-Mercator for a few real networks that fall out of training distribution. Given a real network, we infer the value of parameter β for different dimensions. For instance, a faculty hiring network [1] exhibits $\beta < D$. This could mean that the network resides either in a non-geometric or a weakly-geometric region. Recent work has shown that many real networks are weakly geometric and can be embedded into a hyperbolic disk [2]. However, further work is needed to extend it to higher-dimensional spaces and to explore the interplay between dimensionality and geometricity in complex networks.

In addition, the problem highlighted by the Reviewer is well known in the machine learning community. Domain adaptation addresses the challenge of training a model on one data distribution (in our case, the synthetic networks generated by the $\mathbb{S}^D/\mathbb{H}^{D+1}$ model) and applying it to a related, but distinct, data distribution (the real complex networks) [3, 4]. Our dimensionality–detection task can therefore be viewed as a domain adaptation problem under distribution shift between the synthetic source domain and the real target domain. Although our current method does not include explicit domain adaptation, a natural future extension would be to incorporate an adversarial domain discriminator that attempts to distinguish synthetic from real networks, while the feature extractor is trained to produce domain-invariant representations [5].

Action taken: We added two paragraphs in the Discussion section pointing out the limitations of the current approach as well as possible future work in these directions.

[1] Clauset, A., Arbesman, S. & Larremore D. B. (2015). Systematic inequality and hierarchy in faculty hiring networks. Science Advances 1(1), e1400005.
[2] Van Der Kolk, J., Serrano, M. Á. & Boguñá, M. (2024). Random graphs and real networks with weak geometric coupling. Physical Review Research, 6(1), 013337.
[3] Farahani, A., Voghoei, S., Rasheed, K. & Arabnia, H. R. (2021). A brief review of domain adaptation. Advances in Data Science and Information Engineering: Proceedings from ICDATA 2020 and IKE 2020, 877–894.
[4] Wang, M. & Deng, W. (2018). Deep visual domain adaptation: A survey. Neurocomputing, 312, 135–153.
[5] Tzeng, E., Hoffman, J., Saenko, K. & Darrell, T. (2017). Adversarial discriminative domain adaptation. In Proceedings of the IEEE conference on computer vision and pattern recognition (pp. 7167–7176).

Comment 2.2: Second, while the empirical performance of the extended-persistence features is compelling, the manuscript would benefit from stronger theoretical or mechanistic insight linking the total persistence of chordless cycles to latent dimensionality

The 2022 work offered partial analytical intuition by deriving how small-cycle densities vary with dimension; a similar, even approximate, rationale for the persistence-based descriptors would substantially improve interpretability and clarify the limits of applicability.

Reply: This suggestion has led to a valuable addition to the paper. While an exact analytical relation between latent dimension and the total persistence of chordless cycles is beyond the scope of the present manuscript, we do observe consistent empirical structure that supports a mechanistic interpretation. In Figs. S1 and S2 of the Supplementary Information, we report total persistence values across several

real networks. In these data, higher dimensions tend to be associated with lower total persistence. The trend is not linear and likely depends on additional structural factors, which limits the feasibility of a simple closed-form expression. We highlight this empirical relationship in Fig. R9.

We also evaluated additional persistence-based summaries (e.g., total persistence in dimension 0 and persistence entropy in dimensions 0 and 1), but these features were not sufficiently discriminative for dimension prediction in our setting and were therefore not retained.

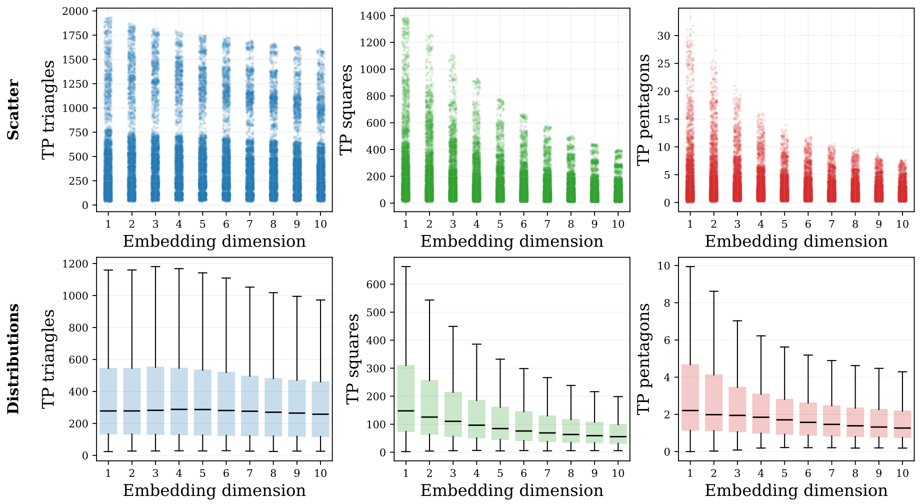

Figure R9: Inverse relationship between network dimension (x-axis) and total extended persistence of cycles (y-axis) for the three filtrations considered: triangles (left), squares (middle), and pentagons (right). *Top row:* Jittered scatter plots; *Bottom row:* Boxplots summarizing the distribution within each dimension. These distributions have been obtained from a sample of 50 000 graphs from the SynNet database, uniformly distributed with respect to dimension.

Action taken: Fig. R9 has ben added to the Supplementary Information as Fig. S3 and has been referenced in page 6 of the main text of the article, including Pearson correlation coefficients.

Comment 2.3: I think, given the broad readership of the journal, moving up Figure 6 (now in the methods section), so that it is in between Figures 1 and 2, or potentially joined with Figure 2, could help the readers the definition of the chordless cycles-based filtration. I understand the narrative choices of the authors, I just think that Figure 6 is just as vital to quickly grasp the foundation of this work as Figures 1 and 2.

Reply: Many thanks for another very helpful comment. Although the primary purpose of the former Fig. 6 was to illustrate our degree-splitting subdivision method, it is true that this figure serves as an enlightening example of what the total extended persistence of cycles is, and how it is computed. Hence, this figure is better placed earlier in the paper, namely in the section where persistence descriptors are defined. Moreover, in order to explain more clearly our method for selecting generating cycles, we have drawn Fig. R10 below, which illustrates the process of filtering by sublevel graphs (yielding the first coordinate of generating cycles), followed by a subsequent filtration by superlevel graphs to find the corresponding second coordinates.

Action taken: The former Fig. 6 in the main body of the paper has been moved and is now Fig. 1. Its caption has been revised to improve clarity and facilitate understanding. Additionally, Fig. R10 has been added to the Supplementary Information as Fig. S4 and is referenced in the main text to provide further insight into the choice of thresholds for the computation of extended persistence.

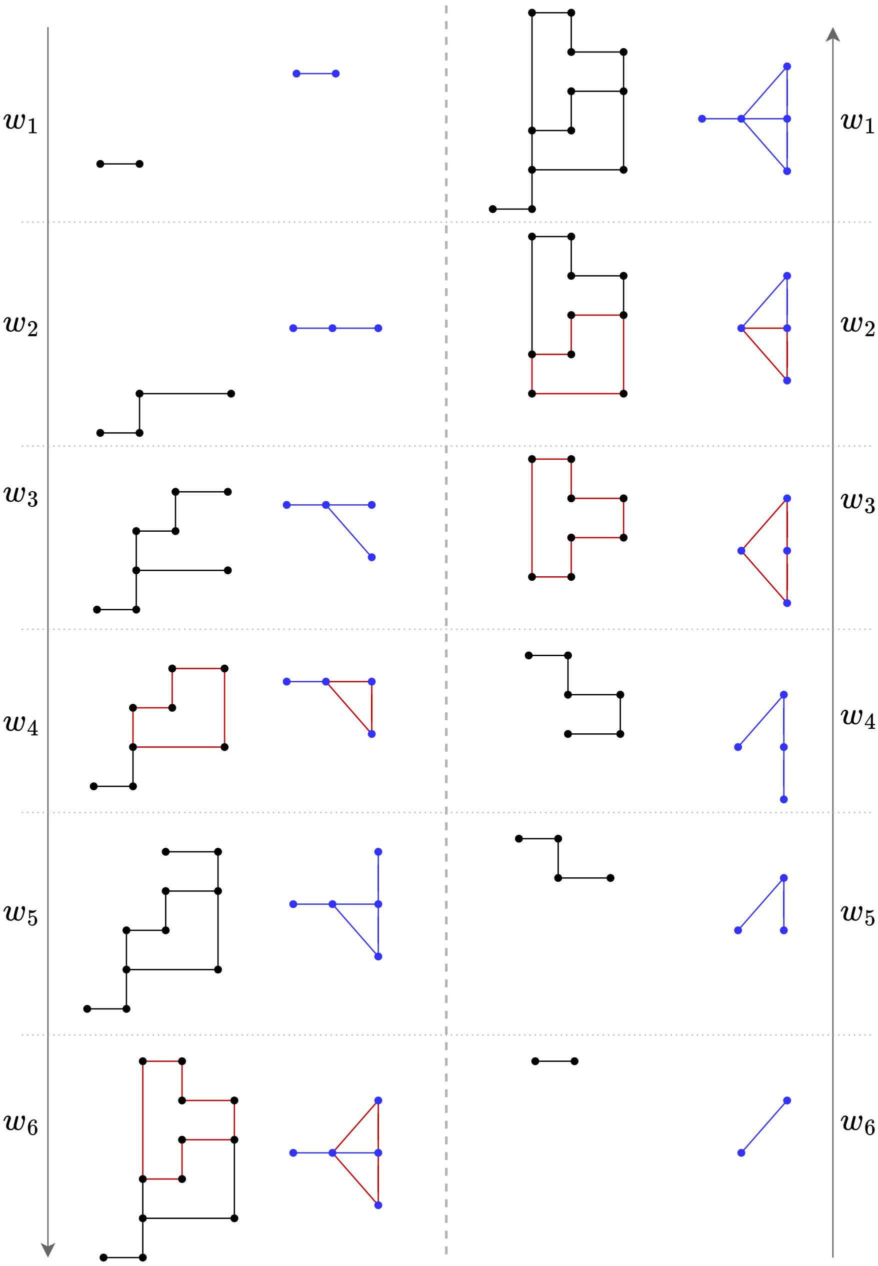

Figure R10: Creation and disappearance of cycles along the sublevel graph filtration (left) followed by the superlevel graph filtration (right) on the example graphs G and G' shown in Fig. 1. The first generating cycle is born at w_4 and dies at w_2 (i.e., it appears at w_2 in the superlevel graph filtration), while the second generating cycle is born at w_6 and dies at w_3. *Blue:* Original edge-weighted graph G; *Black:* Node-weighted graph G' obtained from G by degree-splitting subdivision.

Comment 2.4: In the caption of Table 1, mention that DimNN is the neural network about to be presented in section 2.2.

Reply and action taken: We updated the caption of Table 1 as indicated by the Reviewer.

Comment 2.5: Statistics of the training ensemble might be included in the main text or as supplementary information and mentioned in the main text.

Reply and action taken: We added a table in the Supplementary Information (Table S1) containing the ranges of the features of the SynNet database. This table is now referenced in Section 2.2 (on top of page 12).

Comment 2.6: In conclusion, I congratulate once more with the authors and I believe that this work is to be accepted for publication in the journal upon completion of the improvements suggested.

Reply: We thank the Reviewer once again for their positive feedback and for recommending publication in Nature Communications.

Resubmission of manuscript NCOMMS-25-74043

Chordless cycle filtrations for dimensionality detection in complex networks via topological data analysis

Aina Ferrà Marcús, Robert Jankowski, Meritxell Vila-Miñana, Carles Casacuberta, M. Ángeles Serrano

Reviewer #1

Comment 1.0: I'd like to thank the authors for a thorough revision of their manuscript. They have done a commendable job addressing the previous feedback.

Reply: We thank the Reviewer for their positive and constructive feedback and for recommending publication in Nature Communications.

Reviewer #2

Comment 2.0: I congratulate with the authors for their work. They have successfully addressed all my comments and the improvements to the manuscript now make it a tangible contribution to the field. I recommend this article for publication.

Reply: We thank the Reviewer for their positive and constructive feedback and for recommending publication in Nature Communications.
